# Unveiling the Werner-Type Cluster Chemistry of Heterometallic 4f/Post-Transition Metals: A {Dy_3_Bi_8_} Complex Exhibiting Quantum Tunneling Steps in the Hysteresis Loops and its 1-D Congener

**DOI:** 10.1021/acs.inorgchem.4c04721

**Published:** 2025-01-17

**Authors:** Konstantina H. Baka, Dan Liu, Sagar Paul, Wolfgang Wernsdorfer, Jinkui Tang, Liviu F. Chibotaru, Theocharis C. Stamatatos

**Affiliations:** † Department of Chemistry, University of Patras, Patras 265 04, Greece; ‡ School of Science, Changchun Institute of Technology, Changchun 130012, P. R. China; § Physikalisches Institut, Karlsruhe Institute of Technology (KIT), Kaiserstraße 12, Karlsruhe D-76131, Germany; ∥ Institute for Quantum Materials and Technology (IQMT), Karlsruhe Institute of Technology (KIT), Hermann-von-Helmholtz-Platz 1, Eggenstein-Leopoldshafen D-76344, Germany; ⊥ State Key Laboratory of Rare Earth Resource Utilization, Changchun Institute of Applied Chemistry, Chinese Academy of Sciences, Changchun 130022, P. R. China; # Theory of Nanomaterials Group, Katholieke Universiteit Leuven, Celestijnenlaan 200F, Leuven B-3001, Belgium; ¶ Institute of Chemical Engineering Sciences, Foundation for Research and Technology − Hellas (FORTH/ICE − HT), P.O. Box 1414, Platani, Patras 26504, Greece

## Abstract

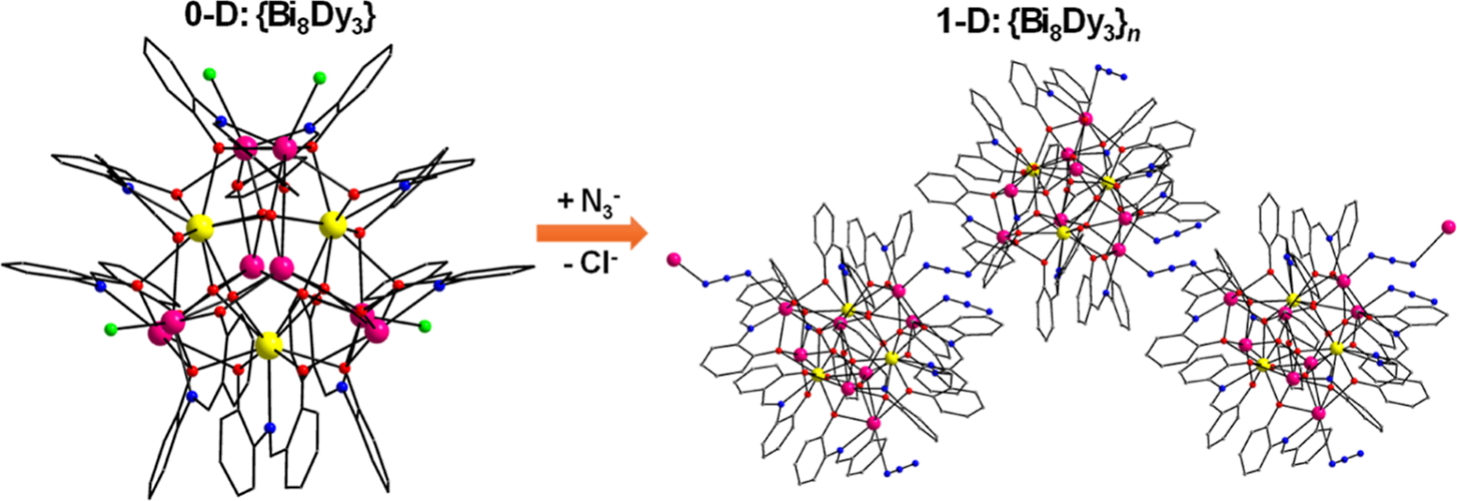

A new [Dy_3_Bi_8_O_6_Cl_3_(saph)_9_] (**1**) Werner-type cluster has been prepared, which is the first Dy^III^/Bi^III^ polynuclear compound with no metal–metal bond and one of the very few Ln^III^–Bi^III^ (Ln = lanthanide) heterometallic complexes reported to date. The molecular compound **1** has been deliberately transformed to its 1-D analogue [Dy_3_Bi_8_O_6_(N_3_)_3_(saph)_9_]_n_ (**2**) via the replacement of the terminal Cl^–^ ions by end-to-end bridging N_3_^–^ groups. The overall metallic skeleton of **1** (and **2**) can be described as consisting of a diamagnetic {Bi_8_} unit with an elongated trigonal bipyramidal topology, surrounded by a magnetic {Dy_3_} equilateral triangle, which does not contain μ_3_-oxo/hydroxo/alkoxo groups. Detailed magnetic studies in a microcrystalline sample and a single crystal of **1** revealed a rare two-step hysteresis loop at various low temperatures and field-sweep rates, with the steps located at zero and ±0.26 T fields providing a measure of intermolecular interactions. Extended ab initio calculations unravel the dominant pathways of magnetization relaxation, as well as the type and magnitude of the magnetic exchange interactions between the Dy^III^ centers and the orientation of their anisotropy axes, thus rendering the {Dy_3_} unit of **1** as a rare triangle among its congeners with a nontoroidal magnetic state. The combined results demonstrate the potential of heterometallic lanthanide/post-transition metal chemistry to provide molecule-based materials with unprecedented structures and compelling methods to rationalize the obtained magnetic properties.

## Abstract 2

The first Dy^III^/Bi^III^ Werner-type cluster [Dy_3_Bi_8_O_6_Cl_3_(saph)_9_] (**1**) with no metal−metal bond has been prepared and subsequently transformed to its 1-D analogue [Dy_3_Bi_8_O_6_(N_3_)_3_(saph)_9_]_n_ (**2**) via the deliberate replacement of the terminal Cl^−^ ions by the end-to-end bridging N_3_^−^ group. Single-crystal hysteresis studies revealed a rare two-step hysteresis loop, while ab initio calculations shed light into the relaxation pathways and the nature of the Dy···Dy exchange interactions.

## 1 Introduction

The synthesis of polynuclear heterometallic complexes bearing paramagnetic 3d-metal ions and lanthanides (Ln) has attracted the interest of inorganic chemists and material scientists over the past few decades, not only because of their fascinating chemical and structural properties^1^ but also due to their contribution to the development of the research fields of molecular magnetism, optics, catalysis, and bioinorganic chemistry.^2^ For instance, it is now established that the magnetic interactions between paramagnetic 3d-metal ions and either anisotropic (i.e., Dy^III^, Tb^III^, Ho^III^, and Er^III^) or isotropic (Gd^III^) 4f-metal ions can be ferromagnetic, thus leading to high-spin molecules with single-molecule magnetic behavior^3^ or magnetocaloric properties,^4^ respectively. On the other hand, the heterometallic chemistry of diamagnetic 3d-metal ions and lanthanides has been barely explored. The vast majority of examples reported to date are limited to the preparation of oligo- and polynuclear Zn^II^/Ln^III^ and Co^III^/Ln^III^ complexes, in which the diamagnetic cations can modulate the electron density distribution of the coordinated organic ligands, through polarization effects, and, consequently, the strength of the crystal field (CF), thus increasing the energy gap between the ground and first excited *m*_J_ states of the anisotropic Ln^III^ ions.^5^

More impressive though is the lack of any previous studies on the heterometallic Werner-type coordination chemistry of paramagnetic and anisotropic Ln^III^ with diamagnetic post-transition metals. This appears to be an appealing subfield of heterometallic chemistry given the rich coordination capabilities of metallic Group 3–5 elements,^6^ along with their diagonal relationship, their unique tendencies in terms of covalent bonding, and the manifestation of inert-pair effect, which is essentially the predilection of the two electrons in the outermost atomic s-orbital to remain nonbonding.^7^ Furthermore, Freedman and co-workers have elegantly proposed and proved the significant effect of spin–orbit coupling (SOC) from heavier diamagnetic post-transition metals on the magnetic anisotropy when binding these elements to first-row transition metals.^8^ This approach focuses on the degree of metal–ligand covalent bonding and the importance of covalency in the transfer of SOC, eventually yielding single-molecule magnets (SMMs) with large magnetic anisotropy and high energy barriers for the magnetization reversal.^9^ SOC arises from the interaction of the two components of a magnetic moment: the spin moment, *S*, and the orbital moment, *L*, and it scales with effective nuclear charge as *Z*_eff_^4^, thereby imposing SOC constants with small values for first-row transition metals compared to their third-row counterparts.^10^ Demir and co-workers have made significant advancements to this field through the preparation of several organometallic and metal–metal-bonded complexes bearing Ln and low-valent bismuth ions, thus yielding unprecedented heterometallic species with impressive SMM properties and open magnetic hysteresis at low temperatures.^11^

Our contribution to this growing field of coordination chemistry is aimed toward the preparation of air-stable, Werner-type heterometallic Ln^III^/Bi^III^ complexes bearing organic chelates which can foster the formation of molecular species with unique structural characteristics and interesting magnetic properties, as a means of exploiting the fascinating chemical and coordination features of both metal ions and the investigation of the effect of diamagnetic Bi^III^ on the electronic properties of the anisotropic and paramagnetic 4f-metal ions. We herein used as a chelate ligand the dianion of the Schiff base ligand *N*-salicylidene-*o*-aminophenol (saphH_2_), which is proved to be capable of binding both trivalent 3d- and 4f-metal ions.^12^ The initial employment of saphH_2_ in heterometallic Dy^III^/Bi^III^ chemistry has led to the unprecedented, endecanuclear [Dy_3_Bi_8_O_6_Cl_3_(saph)_9_] (**1**) molecular (0-D) cluster, exhibiting slow relaxation of magnetization and magnetic hysteresis loops with periodic steps due to quantum tunneling of magnetization (QTM). Extended ab initio calculations have been carried out to shed light on the dominant pathways of magnetization relaxation, as well as the nature and magnitude of the exchange interactions between the Dy^III^ centers and the orientation of their anisotropy axes. Furthermore, exploiting the “structurally exposed”, terminally bound, Cl^–^ groups, we have been able to deliberately replace them with end-to-end bridging N_3_^–^ groups, thus facilitating the formation of the polymeric analogue [Dy_3_Bi_8_O_6_(N_3_)_3_(saph)_9_]_*n*_ (**2**) consisting of a 1-D zigzag chain of chemically intact {Dy_3_Bi_8_O_6_(saph)_9_} repeating units.

## 2 Experimental Section

### 2.1 Synthesis

Aerobic conditions were applied to all manipulations using materials (reagent grade) and solvents as received, unless otherwise noted. The Schiff base ligand saphH_2_ was prepared, purified, and characterized as described elsewhere.^12^*Caution!* Azide salts, and their corresponding metal complexes, are potentially explosive; such compounds should be synthesized and used in small quantities and always treated with utmost care. Compound **2** does not detonate on shock or spark under the reported synthetic methods and conditions.

#### 2.1.1 [Dy_3_Bi_8_O_6_Cl_3_(saph)_9_]

To a stirred, orange solution of saphH_2_ (0.04 g, 0.20 mmol) and NEt_3_ (28 μL, 0.20 mmol) in a solvent mixture comprising MeOH/MeCN (15 mL, 2:1 v/v) were added simultaneously solids BiCl_3_ (0.06 g, 0.20 mmol) and DyCl_3_·6H_2_O (0.08 g, 0.20 mmol). The resulting orange suspension was stirred for 40 min, during which time all of the solids were dissolved. The solution was then filtered, and the filtrate was allowed to evaporate slowly at room temperature. After 4 days, X-ray quality yellow plate-like crystals of **1** appeared, and these were collected by filtration, washed with cold MeOH (2 × 2 mL) and Et_2_O (2 × 5 mL), and dried in air for 24 h. The yield was 45% (based on saphH_2_). The dried crystalline solid was analyzed as **1.** Anal. calc. for C_117_H_81_Dy_3_Bi_8_N_9_O_24_Cl_3_ (found values in parentheses): C 32.97% (32.82%), H 1.92% (1.85%), and N 2.96% (3.09%). Selected IR data (KBr, cm^–1^): 3421 (mb), 1608 (vs), 1583 (m), 1543 (m), 1472 (vs), 1439 (m), 1386 (m), 1285 (s), 1251 (m), 1173 (w), 1151 (m), 1122 (m), 1036 (w), 969 (w), 916 (m), 866 (w), 826 (m), 749 (s), 654 (w), 600 (m), 491 (mb), 442 (w), 424 (w).

#### 2.1.2 [Dy_3_Bi_8_O_6_(N_3_)_3_(saph)_9_]_*n*_

##### 2.1.2.1 Method A

To a stirred, yellow suspension of microcrystalline complex **1** (0.43 g, 0.10 mmol) in a solvent mixture comprising MeCN/THF (15 mL, 1:2 v/v) was added dropwise Me_3_SiN_3_ (54 μL, 0.40 mmol) over a period of 20 min, during which time the microcrystalline solid was dissolved. The resulting yellow solution was filtered, and the filtrate was left to evaporate slowly at room temperature. After 3 days, X-ray quality yellow plate-like crystals of **2** appeared, and these were collected by filtration, washed with cold MeCN (2 × 2 mL) and Et_2_O (2 × 5 mL), and dried in air for 24 h. The yield was 20% (based on compound **1**). The dried crystalline solid was analyzed as **2.** Anal. calc. for C_117_H_81_Dy_3_Bi_8_N_18_O_24_ (found values in parentheses): C 32.82% (32.95%), H 1.91% (2.05%), and N 5.89% (5.77%). Selected IR data (KBr, cm^–1^): 3425 (mb), 2016 (m), 1610 (vs), 1583 (m), 1544 (m), 1471 (vs), 1439 (m), 1384 (m), 1288 (s), 1258 (m), 1172 (w), 1151 (m), 1122 (m), 1036 (w), 970 (w), 917 (m), 866 (w), 827 (m), 748 (s), 600 (m), 551 (w), 491 (mb), 444 (w), 423 (w).

##### 2.1.2.2 Method B

To a stirred orange solution of saphH_2_ (0.04 g, 0.20 mmol) and NEt_3_ (28 μL, 0.20 mmol) in a solvent mixture comprising MeOH/MeCN (15 mL, 2:1 v/v) was added Me_3_SiN_3_ (108 μL, 0.80 mmol). To the resulting orange solution were added simultaneously solids BiCl_3_ (0.06 g, 0.20 mmol) and DyCl_3_·6H_2_O (0.08 g, 0.20 mmol) without any noticeable color change. The orange solution was stirred for 40 min and then filtered, and the filtrate was left to evaporate slowly at room temperature. After 5 days, X-ray quality yellow plate-like crystals of **2** appeared, and these were collected by filtration, washed with cold MeOH (2 × 2 mL) and Et_2_O (2 × 5 mL), and dried in air for 24 h. The yield was 52% (based on saphH_2_). The identity of the crystalline material was confirmed by IR spectroscopic comparison with the crystals of Method A and CHN elemental analyses.

### 2.2 X-ray Crystallography

Single-crystal X-ray diffraction data of complexes **1** and **2** were collected on yellow block-like crystals [0.03 × 0.02 × 0.01 mm (**1**) and 0.12 × 0.10 × 0.10 mm (**2**)] using a Rigaku Oxford Diffraction XtaLAB Synergy diffractometer equipped with a HyPix-6000HE area detector at 173 K and utilizing Cu Kα (λ = 1.54184 Å) radiation from a PhotonJet microfocus X-ray source. The structures were solved using SHELXT ver. 2018/2^13^ and refined by full-matrix least-squares techniques against *F*_0_^2^ using the SHELXL ver. 2018/3^14^ program through the OLEX2 interface.^15^ The non-hydrogen atoms were successfully refined using anisotropic displacement parameters, and hydrogen atoms bonded to the carbon and oxygen atoms of the coordinated ligands were placed at their idealized positions using appropriate *HFIX* instructions in SHELXL. All these atoms were included in subsequent refinement cycles in riding-motion approximation with isotropic thermal displacement parameters (*U*_iso_) fixed at 1.2 or 1.5 × *U*_eq_ of the relative atom.

Various figures of the structures were created, using Mercury^16^ and Diamond^17^ software packages. The unit cell parameters, structure solution, and refinement details of **1** and **2** are summarized in Table 1. Further crystallographic details can be found in the corresponding CIF files provided in the Supporting Information.

**Table 1:** Crystal Data and Structural Refinement Parameters for Compounds **1** and **2**

identification code	**1**	**2**
empirical formula	C_117_H_81_Bi_8_Dy_3_N_9_O_24_Cl_3_	C_117_H_81_Bi_8_Dy_3_N_18_O_24_
formula weight/g mol^–1^	4262.59	4282.33
temperature/K	173.0	173.0
crystal system	triclinic	monoclinic
space group	*P*1̅	*P*2_1_/*c*
*a*/Å	16.6121(3)	20.9925(3)
*b*/Å	17.1652(3)	23.5891(2)
*c*/Å	26.2397(5)	28.3147(3)
α/deg	87.078(1)	90
β/deg	78.601(2)	97.243(1)
γ/deg	63.643(2)	90
volume/Å^3^	6566.4(2)	13909.4(3)
*Z*	2	4
ρ_calc_/g cm^–3^	2.174	2.045
μ/mm^–1^	30.820	28.426
F(000)	3936	7852
radiation	Cu Kα	Cu Kα
	λ = 1.54184	λ = 1.54184
θ range/deg	2.831–75.957	2.794–73.687
index ranges	–19 ≤ *h* ≤ 19	–25 ≤ *h* ≤ 25
	–20 ≤ *k* ≤ 20	–24 ≤ *k* ≤ 28
	–31 ≤ *l* ≤ 31	–33 ≤ *l* ≤ 33
reflections collected	69817	84508
independent reflections	19029 (*R*_int_ = 0.0607)	18344 (*R*_int_ = 0.0783)
goodness-of-fit on F^2^	1.075	1.061
final *R*^a^^,^^b^ indices [*I* > 2σ(*I*)]	*R*_1_ = 0.0468; *wR*_2_ = 0.1208	*R*_1_ = 0.0588; *wR*_2_ = 0.1645
final *R*^a^^,^^b^ indices [all data]	*R*_1_ = 0.0561; *wR*_2_ = 0.1257	*R*_1_ = 0.0747; *wR*_2_ = 0.1751
(Δρ)_max,min_/e Å^–3^	2.850 and −1.887	3.982 and −2.131
CCDC number	2380490	2380491

a *R*_1_ = Σ(||*F*_*o*_| – |*F*_*c*_||)/Σ|*F*_*o*_|.

b *wR*_2_ = [Σ[*w*(*F*_0_^2^ – *F*_c_^2^)^2^]/Σ[*w*(*F*_0_^2^)^2^]]^1/2^, *w* = 1/[σ^2^(*F*_0_^2^) + (*ap*)^2^ + *bp*], where *p* = [max(*F*_0_^2^, 0)+ 2*F*_c_^2^]/3.

### 2.3 Physical Measurements

Elemental analyses (C, H, and N) were performed by the University of Patras microanalytical service. Infrared (IR) spectra (4000–400 cm^–1^) were recorded in the solid state using a PerkinElmer 16 PC spectrometer with samples prepared as KBr pellets (Figure S1). Powder X-ray diffraction (p-XRD) measurements were conducted on a Bruker D8 ADVANCE X-ray diffractometer using Cu–Kα radiation. Magnetic susceptibility studies were performed in the temperature range 1.9–300 K using a Quantum Design MPMS XL-7 SQUID magnetometer equipped with a 7 T magnet. The direct current (dc) magnetic susceptibility measurements were performed with an external magnetic field of 1000 Oe in the temperature range 1.9–300 K, and the alternating current (ac) measurements were measured in a 3.0 Oe ac field oscillating at different frequencies from 1 to 1000 Hz. The experimental magnetic susceptibility data were corrected for the diamagnetism estimated from Pascal’s tables and sample holder calibration.^18^ Low-temperature (30 mK – 5.0 K) magnetization (*M*) versus field (*H*) measurements were performed on a single crystal using an array of μ-SQUIDs inside a dilution refrigerator equipped with a 3D vector magnet.^19^ The high sensitivity of this magnetometer allows the study of single crystals of SMMs of the order 10–500 μm. The field can be applied in any direction with a precision better than 0.1° by separately driving three orthogonal coils. Crystals of **1** were maintained in mother liquor to avoid degradation and were covered in Apiezon grease for protection during the transfer to the μ-SQUID and thermalization during subsequent cooling.

### 2.4 Computational Studies

First, the BP DKH-def2-SVP (SARC-DKH-TZVP for Bi and Gd atoms replacing Dy) method was used to obtain the Mulliken charge of **1** by the ORCA software.^20^ Then, the Dy-fragment ab initio calculation was carried out on the OpenMOLCAS and main part including the 9-coordinate Dy^III^ spin center and the other two Dy^III^ ions replaced by closed-shell Lu^III^ ions.^21^ The other atoms were set as point charge from DFT Mulliken charge.^22^ The relevant basis set of the main part is shown in Table S1. Coordinates were extracted from the X-ray crystal structure. Relativistic effects were treated in two steps on the basis of the Douglas–Kroll Hamiltonian.^23^ First, the scalar terms were included in the basis set generation and used to determine the spin-free wave functions and energies in the complete active space self-consistent field (CASSCF) method.^24^ Next, SOC was added within the restricted active space state interaction (RASSI-SO) method, which uses the spin-free wave functions as basis states.^25^ Active space of the CASSCF method included 9 electrons in 7 orbitals for Dy^3+^ (4f^9^ configuration). State-averaged CASSCF calculations were performed for all sextets (21 states), all quadruplets (224 states), and all doublets (490 states) of the Dy^III^ ions.^26^ We have mixed the maximum number of spin-free states, which was possible with our hardware. To this end, 21 sextets, 128 quadruplets, and 130 doublets were mixed through SOC in RASSI-SO. On the basis of the resulting spin–orbital multiplets, SINGLE_ANISO program was used to compute the local magnetic properties (*g*-tensors, main magnetic axes, magnetization blocking barrier, etc.).^27^ The magnetic properties of the entire complex **1**, involving three Dy(III) centers, were calculated by the POLY_ANISO routine,^27,28^ in which the anisotropic exchange interactions were simulated within the Lines model.^29^

## 3 Results and Discussion

### 3.1 Synthetic Comments

Our research group has been actively exploring reaction systems that include the utilization of various Schiff base ligands in 3d, 4f, or mixed 3d/4f metal cluster chemistry, targeting at the isolation of new compounds with unprecedented structural motifs and potentially interesting magnetic and/or optical properties.^30^ Among other Schiff base ligands, particularly appealing to us are those that resemble the scaffolds of saphH_2_ and *N*-naphthalidene-*o*-aminophenol (naphH_2_), both possessing a relatively soft N atom and two hard, upon double deprotonation, O atoms that can bind to a single or multiple metal centers. Indeed, the employment of the saphH_2_ chelate in heterometallic Mn/Dy chemistry has afforded two novel complexes, {Mn^III^_4_Dy_5_} and {Mn^III^_4_Dy_3_}, with new topologies and SMM behavior.^12^ Furthermore, the use of the naphH_2_ ligand, which exhibits similar chemical and electronic properties with saphH_2_ but it is bulkier and sterically more rigid, in Mn/Dy and Co/Ln chemistry has recently yielded two {Mn^III^_2_Dy_2_} complexes possessing linear or zigzag metal core topologies,^31^ as well as a family of triangular {Co^III^_2_Ln} clusters exhibiting slow magnetization relaxation.^32^

As a part of our recent research efforts toward the systematic study of heterometallic 4f/post-transition metal chemistry, we decided to employ the saphH_2_ chelate as a means of obtaining new Ln^III^/Bi^III^ cluster compounds with novel structures and intriguing magnetic properties. To this end, the 1:1:1:1 reaction between BiCl_3_, DyCl_3_·6H_2_O, saphH_2_, and NEt_3_, in a solvent mixture comprising MeOH and MeCN, afforded yellow crystals of the heterometallic cluster **1** in yields as high as 45% upon slow evaporation at room temperature. Interestingly, when carrying out the aforementioned experimental reaction but using amounts of the starting reagents according to stoichiometric eq 1, complex **1** was isolated as a microcrystalline solid in yields as high as 80%; the identity of the microcrystalline solid was established through elemental analysis studies and comparison of the IR spectrum with that of authentic crystals of **1**
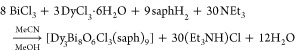
1

The solvent mixture of MeOH/MeCN was proved to be a decisive factor for the preparation of crystalline and pure compound **1**. The solvent MeOH facilitates the dissolution of all starting materials (metal salts, organic ligand, and base) and it also keeps soluble the (Et_3_NH)Cl byproduct, while the solvent MeCN appears to assist toward the growth of crystals of **1** (vide infra). In addition, the base NEt_3_ was also found to be important for the formation of **1**, most likely due to its ability to deprotonate (in the presence of metal ions) the saphH_2_ chelate and the H_2_O molecules in solution, thus generating the coordinated saph^2–^ and O^2–^ groups, respectively. In the absence of a base, no solid-state compounds were detected over the course of two months upon the application of several crystallization techniques, suggesting the presence of different compounds in the corresponding solutions.

The presence of terminally and weakly bound Cl^–^ ions in the coordination sphere of some Bi^III^ atoms sparked our interest in attempting to deliberately replace them by other ions with the ability to link the {Dy_3_Bi_8_} clusters into multidimensional coordination polymers. To this direction, end-to-end azide (N_3_^–^) seemed a potentially promising group to facilitate the aggregation of cluster **1** into a polymer-of-cluster motif. Indeed, the reaction of microcrystalline **1** with an excess of Me_3_SiN_3_ in a solvent mixture comprising MeCN/THF yielded yellowish crystals of the 1-D compound **2** in 20% yield. The general formation of **2** is summarized by the following stoichiometric equation, eq 2
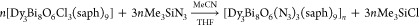
2

The co-solvent THF was used due to the excellent solubility of cluster **1**, whereas MeCN was proved to be the most appropriate solvent for the crystallization of the resulting product **2**. Increase of the overall yield of **2** to 52% was achieved through the 1:1:1:1 self-assembly reaction between BiCl_3_, DyCl_3_·6H_2_O, saphH_2_, and NEt_3_, in the presence of 4 equiv of Me_3_SiN_3_, in a solvent mixture of MeOH/MeCN. The structural and chemical features of **2** corroborate the preference of Bi^III^ atoms for binding with the N_3_^–^ groups rather than the Cl^–^ ions, regardless of the presence of both ions in the reaction solution.^33^

The IR spectra of both compounds (Figure S1) are very similar to each other, thus corroborating their similar structural characteristics. In both compounds, the very strong band at ∼1610 cm^–1^ is assigned to the C=N stretching vibration of the Schiff base linkage, ν(C=N). This band has been shifted to lower frequencies on going from the free ligand saphH_2_ [ν(C=N) = 1625 cm^–1^] to complexes **1** and **2**; this is due to the coordination of the imino N atom to the metal centers.^34^ The band at ∼1285 cm^–1^ is associated with phenolate-type C–O stretching vibrations.^34^ In the IR spectrum of complex **2**, the medium intensity band at 2016 cm^–1^ is assigned to the asymmetric stretching mode of the end-to-end bridging azido ligands. As expected, this band does not appear in the IR spectrum of **1**.^30^

### 3.2 Description of Structures

**Figure 1:**
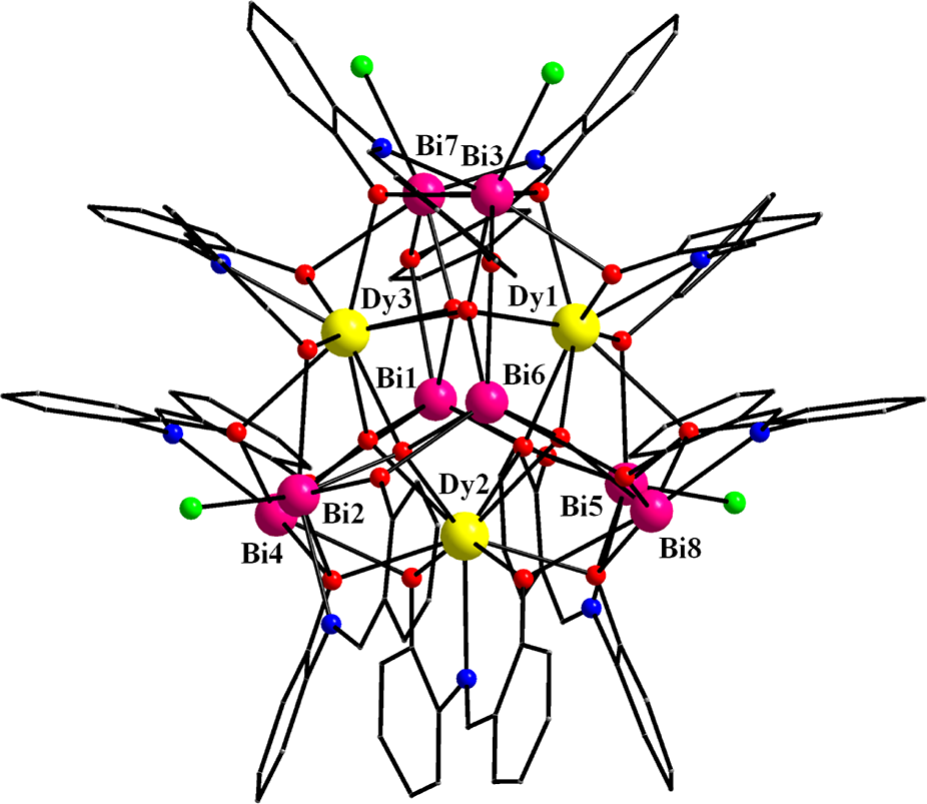
Partially labeled representation of complex **1**. Color scheme: Dy^III^, yellow; Bi^III^, magenta; Cl, green; O, red; N, blue; C, gray. H atoms are omitted for clarity.

**Figure 2:**
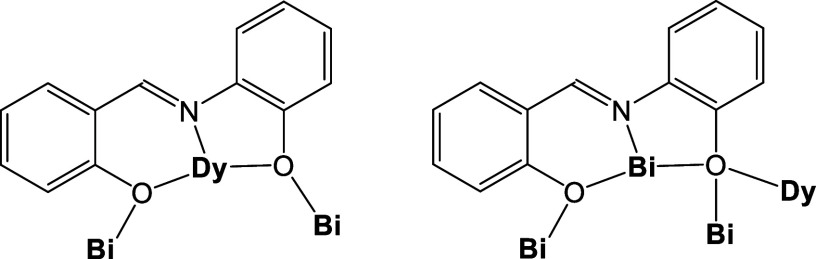
Crystallographically established coordination modes of saph^2–^ ligands present in complexes **1** and **2**.

**Figure 3:**
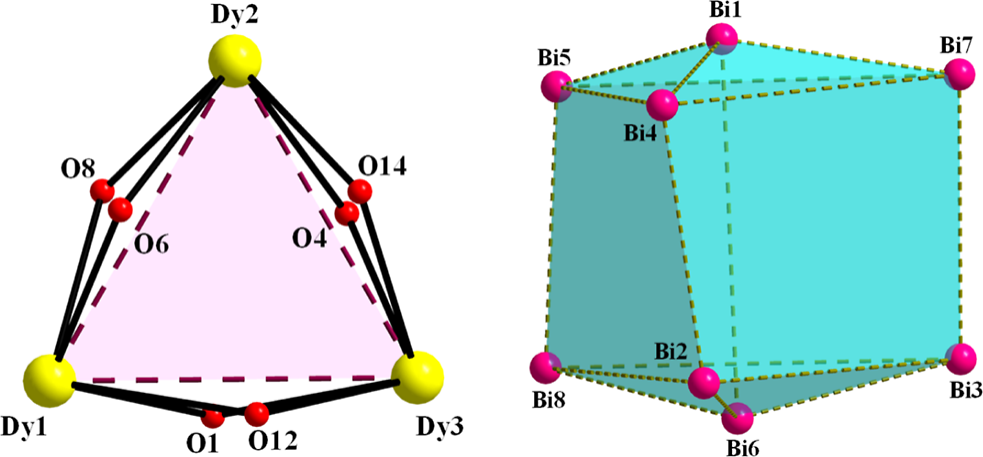
Representation of (left) the {Dy_3_(μ–OR)_6_}^3+^ triangular subunit, and (right) the elongated trigonal bipyramidal topology of the {Bi_8_} subunit within **1**. The dashed lines are virtual bonds to emphasize the corresponding polyhedra (triangles and squares). Color scheme as in Figure 1.

A partially labeled structure of neutral complex **1** is shown in Figure 1. Selected interatomic distances and angles for complexes **1** and **2** are listed in Tables S2 and S3, respectively. Compound **1** crystallizes in the triclinic space group *P*1̅ with the heterometallic {Dy_3_Bi_8_} cluster in a general position. The molecular structure of **1** consists of three Dy^III^ and eight Bi^III^ atoms bridged together through six μ_4_–Ο^2–^ ions and the phenoxido groups (μ–OR^–^ and μ_3_–OR^–^) of six η^2^:η:^1^η:^3^μ_4_ and three η^2^:η:^1^η:^2^μ_3_ saph^2–^ ligands, respectively (Figure 2). The structure can be described as six {Dy_2_Bi_2_(μ_4_–Ο^2–^)}^10+^ edge-sharing tetrahedra that share common Dy···Bi edges with their neighboring units. The μ–OR^–^ and μ_3_–OR^–^ groups serve to link Dy···Bi and Bi···Bi pairs, while the only linkage between the Dy···Dy pairs is provided by the μ–O^2–^ ions (as a part of their overall μ_4_-bridging capacity). Therefore, the overall core of the compound is {Dy_3_Bi_8_(μ_4_–Ο)_6_(μ_3_–OR)_5_(μ–OR)_13_}^3+^ (Figure S2) and it can be alternatively described as a central, near equilateral {Dy_3_} triangle surrounded by a {Bi_8_} subunit with a distorted elongated trigonal bipyramidal topology (Figure 3), as confirmed by the SHAPE program (CShM = 5.34);^35^ the second closest geometry for the {Bi_8_} metallic skeleton is that of a very distorted cube with a CShM value of 17.58. Peripheral ligation about the core is provided by the chelating parts of the nine doubly deprotonated saph^2–^ ligands, as well as four terminally bound Cl^–^ (on Bi2, Bi3, Bi5, and Bi7) from which one (Cl1) is 100% occupancy, while the other three Cl^–^ ions occupy each site with a two-third occupancy over the entire position to yield an overall three Cl^–^ ions per cluster compound.

The three Dy^III^ atoms within the triangular subunit are held together through six μ–Ο^2–^ (O1, O4, O6, O8, O12, and O14), two of them on each Dy···Dy edge (Figure 3, left), rendering it the first {Dy_3_} triangle with no central μ_3_-bridging group reported to date in the literature. The triangular metallic core is nearly equilateral with the three Dy···Dy edges (Dy1–Dy2 = 4.139 Å, Dy2–Dy3 = 4.141 Å, and Dy3–Dy1 = 4.084 Å) being almost equal within the usual 3σ criterion. Furthermore, the Dy1···Dy2···Dy3, Dy2···Dy3···Dy1, and Dy3···Dy1···Dy2 angles of the metallic {Dy_3_} triangle are 59.1°, 60.4°, and 60.5°, respectively, confirming its equilateral conformation. The Dy–O(R)–Dy angles are very close in values, and they fall into the range 109.3(2)–112.1(3)°. On the other hand, the elongated trigonal bipyramidal topology of the {Bi_8_} unit is composed of six {Bi_3_} triangles, with three of them on the two opposite sides (Bi1–Bi4–Bi5, Bi1–Bi4–Bi7, Bi1–Bi5–Bi7 and Bi6–Bi2–Bi8, Bi6–Bi2–Bi3, Bi6–Bi8–Bi3), and three {Bi_4_} squares (Bi4–Bi5–Bi8–Bi2, Bi2–Bi3–Bi7–Bi4, Bi3–Bi7–Bi5–Bi8); the latter deviate significantly from the ideal square due to the unequal Bi···Bi sides (Figure 3, right). All of the Bi^III^ atoms are linked to each other through μ–OR^–^ and μ–Ο^2–^ bridging groups.

All three Dy^III^ atoms are 9-coordinate with a slightly distorted spherical capped square antiprismatic geometry, as established by the SHAPE program [CShM = 1.51 (Dy1), 1.15 (Dy2), and 1.36 (Dy3), Figure S3 and Table S4]. In all cases, the eight O-donor atoms from the O^2–^ and saph^2–^ ligands define the square antiprism, with the Dy^III^ atom located at the center, while the capping site is occupied by the imine *N*-donor atom of a saph^2–^ ligand. In contrast, the eight Bi^III^ atoms in **1** are six- and seven-coordinate, adopting three different geometries according to the CShM values of the SHAPE program (Figure S3 and Table S5). The six-coordination revealed vacant coordination sites for the corresponding Bi^III^ atoms, which is a first indication for the arrangement of the lone pair of 6s electrons. The space-filling plot (Figure S4) revealed the exposure of the terminally bound Cl^–^ ions, occupying the peripheral sites of the cluster compound, as well as the nanosized dimensions of the “bowl”-shaped **1**, with the longest intramolecular C···C distance being ∼17 Å, excluding the H atoms. The {Dy_3_Bi_8_} clusters interact with each other in the crystal through: (i) weak CH···π stacking interactions between the phenyl rings of the coordinated saph^2–^ ligands and (ii) weak interactions between the aromatic rings of saph^2–^ and the lone pair of electrons at some vacated Bi^III^ atoms. Hence, the shortest Dy···Dy intermolecular distance is 12.202(1) Å (Figure S5), presaging negligible intermolecular magnetic interactions between neighboring {Dy_3_Bi_8_} clusters in the crystal.

**Figure 4:**
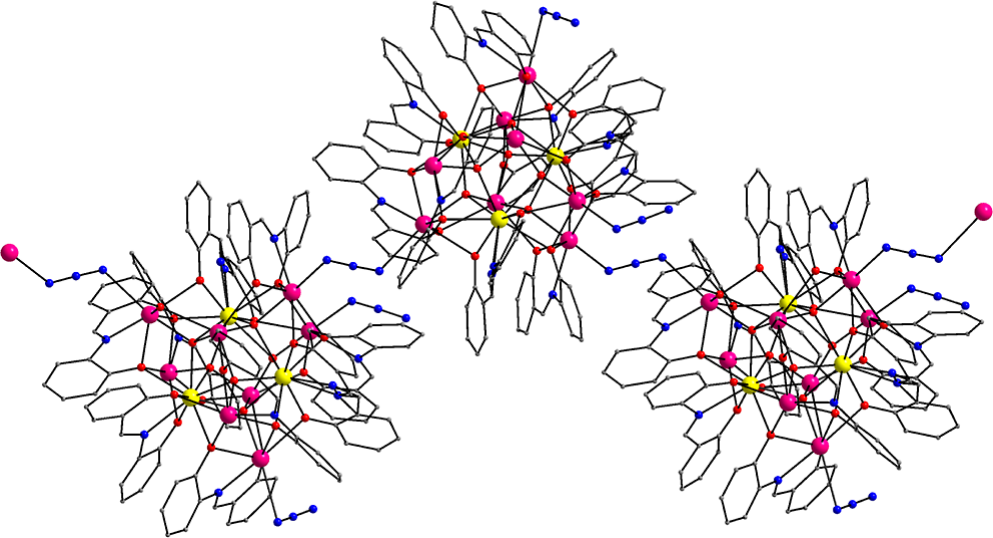
Small portion of the 1-D zigzag polymeric structure of **2**. Color scheme: Dy^III^, yellow; Bi^III^, magenta; O, red; N, blue; C, gray. H atoms are omitted for clarity.

Compound **2** is a 1-D polymeric analogue of molecular cluster **1** and has been derived from the replacement of the terminally coordinated Cl^–^ ions in **1** by the respective number of azides (N_3_^–^). Two of the N_3_^–^ groups in **2** act as end-to-end (μ-1,3 or 2.11) bridging ligands, providing a covalent linking of the {Dy_3_Bi_8_} clusters into an overall one-dimensional zigzag topology, which is expanded along the crystallographic *c*-axis (Figures 4 and S6). There are negligibly other structural or chemical differences between **1** and **2** related to the connectivity of the metal ions with the binding ligands or the coordination geometries of the Bi^III^ and Dy^III^ atoms. From the supramolecular viewpoint, the 1-D chains of **2** are weakly interacting with their neighboring units through CH···π stacking interactions between the phenyl rings of the coordinated saph^2–^ ligands (Figure S7), whereas the shortest Dy···Dy intercluster distance is 12.390(2) Å.

Finally, it is worthy to mention that there is only a family of Bi/Ln (Ln = Eu, Y, Pr, and Sm) clusters reported to date, all with different nuclearities and topologies, and these have been prepared by the employment of *tert*-butylphosphonic acid and β-diketones as bridging and capping ligands.^36^ However, none of these heterometallic compounds contained Dy^III^ atoms, and none of these were magnetically studied.

### 3.3 Magnetic Studies

**Figure 5:**
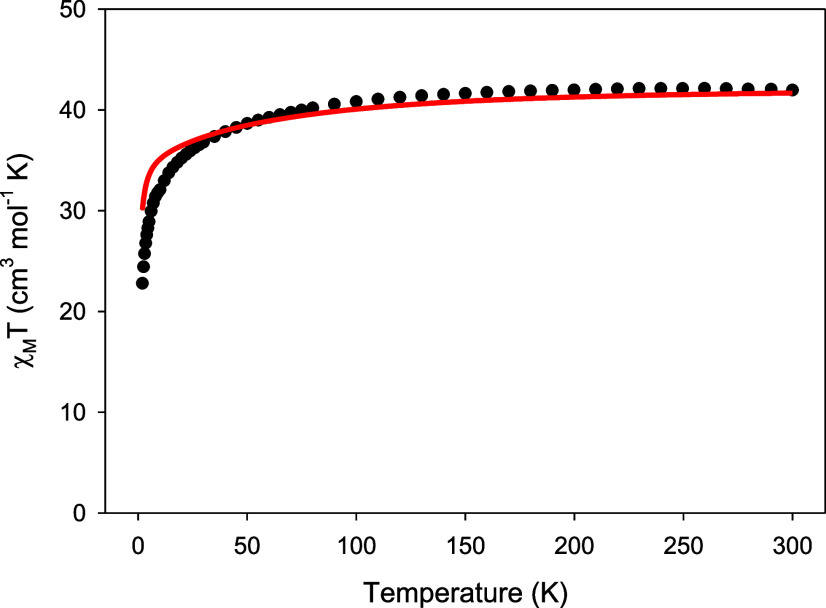
Temperature dependence of the χ_Μ_*T* product for complex **1** at 0.1 T. The solid red line corresponds to the curve generated from the ab initio studies.

Although complexes **1** and **2** differ in their overall topology (0-D vs 1-D), their magnetic properties were proved to be similar; this is not surprising given the fact that the {Dy_3_Bi_8_} clusters are chemically the same and the supramolecular features do not differentiate significantly. Thus, we will restrict the analysis of the magnetic properties and dynamics on molecular compound **1**, while the magnetic response of **2** is presented in Figures S8 and S9. Direct current (dc) magnetic susceptibility measurements were carried out on a microcrystalline sample of analytically pure **1** (as derived by elemental analysis studies) in the 2–300 K range under an applied magnetic field of 0.1 T (Figure 5). The p-XRD patterns of both compounds **1** and **2** show good agreement with the simulated ones, confirming the phase purity of the samples (Figures S10 and S11). The room temperature χ_Μ_*T* value of 41.97 cm^3^ K mol^–1^ is very close to the theoretical value of 42.51 cm^3^ K mol^–1^ for three noninteracting Dy^III^ ions (^6^H_15/2_, *S* = 5/2, *L* = 5, and *g* = 4/3). The χ_Μ_*T* product remains almost constant until ∼170 K, then it decreases smoothly upon further cooling until ∼30 K, and more sharply at the end, reaching a value of 22.79 cm^3^ K mol^–1^ at 2 K. The rapid decline of the χ_Μ_*T* product upon lowering the temperature is mostly due to the depopulation of the CF *m*_J_ microstates and the onset of some weak intramolecular antiferromagnetic interactions between the three Dy^III^ ions.^37,38c^

**Figure 6:**
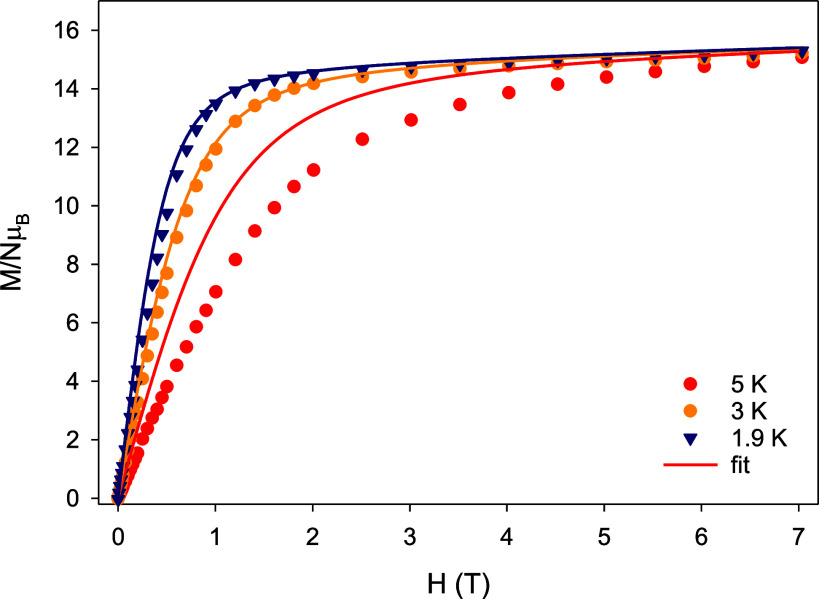
Plots of magnetization (*M*) vs field (*H*) for complex **1** at three different low temperatures. The solid lines represent the calculated curves obtained by ab initio calculations.

The dependence of the magnetization (*M*) upon the external magnetic field (*H*) at temperatures of 1.9, 3, and 5 K reveals a relatively abrupt increase at low fields without reaching saturation at 7 T (Figure 6); this behavior is indicative of the presence of magnetic anisotropy. Moreover, the magnetization value at 7 T is ∼15.3 *N*μ_B_, much lower than the expected value of magnetization saturation (*M*_S_) for three Dy^III^ ions (*M*_S_/*N*μ_B_ = n*g*_*J*_*J* = 30 *N*μ_B_ for *n* = 3, *g*_J_ = 4/3, and *J* = 15/2); this behavior is ascribed to the CF effects that induce the overall magnetic anisotropy of the system.^38^ The absence of any clear signs of *S*-shaped *M*(*H*) curves at low fields is a first indication that the {Dy_3_} unit in **1** will not exhibit a single-molecule toroidal behavior, in contrast to some previously reported μ_3_–Ο^2–^/OH^–^/OR^–^-bridged {Dy_3_} triangles.^39^ A similar response has been observed for the homo- and heterometallic compounds [Dy_3_(L)(μ_3_–ΟMe)_2_(NO_3_)_3_]^+^ and [Zn_3_Dy_3_(μ_6_–CO_3_)(μ_3_–OH)_3_(L′)_3_(H_2_O)_3_]·3(ClO_4_)·(NO_3_), respectively, where L is a multidentate amino-bis(phenolate) ligand and L′ is 6,6′-{[2-(dimethylamino)ethylazanediyl]bis(methylene)}bis(2-meth-oxy-4-methylphenol).^40^

**Figure 7:**
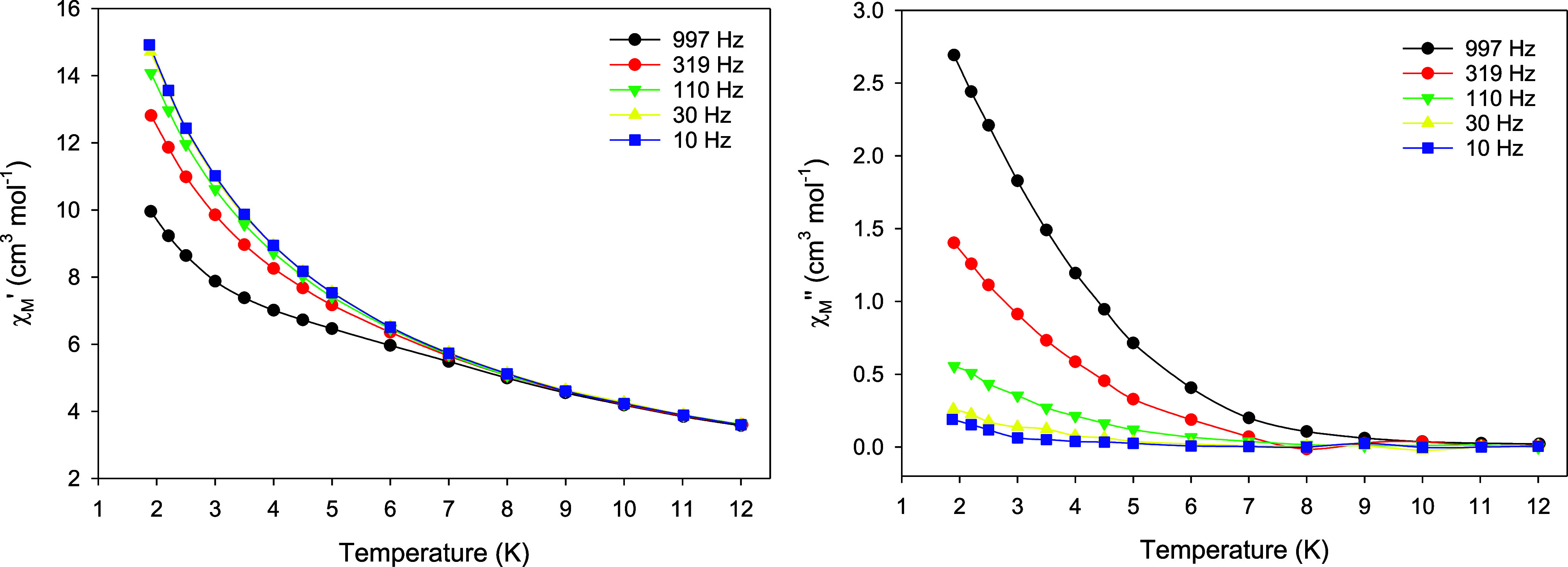
Temperature dependence of the in-phase (χ_Μ_′, left) and out-of-phase (χ_Μ_″, right) ac magnetic susceptibilities at the 600 Oe dc field for **1**, measured in a 3.0 G ac field oscillating at the indicated frequencies. The solid lines are guides only.

The magnetic dynamics of complex **1** were initially studied through alternating current (ac) magnetic susceptibility measurements at a zero applied dc field under a weak ac field of 3.0 G oscillating at the frequency of 1000 Hz. The compound showed a frequency-dependent tail of peak in the out-of-phase (χ_Μ_″) susceptibility vs T plot at a temperature below ∼9 K, suggesting the onset of slow magnetization relaxation and possible SMM behavior (Figure S12). This is not unusual in polynuclear 4f-based SMMs with low symmetry structures, in which the metal ions are characterized by various distorted coordination geometries with a random projection of their single-ion anisotropies along the molecular easy-axis.^41^ Specifically, for Kramers ions, such as Dy^III^, in most coordination environments, the presence of transverse anisotropy, dipole–dipole interactions, and hyperfine interactions facilitates the mixing of the individual Dy^III^ ground states in a zero dc field, thereby enhancing the QTM mechanism over thermally assisted relaxation processes.^42^ To increase the magnetization blocking, an external optimum dc field is usually applied to the ac magnetometry, aiming at the shift of the χ_Μ_″ signals at higher temperatures and the observation of entirely visible peaks.^43^ From the peak maximum in the diagram of χ_Μ_″ vs *H*_dc_ at the lowest possible temperature of 1.9 K and a fixed ac frequency of 1000 Hz, we were able to deduce an optimum *H*_dc_ of 600 Oe (Figure S13). Subsequently, ac studies under an *H*_dc_ of 600 Oe were carried out but, still, only frequency-dependent tails of peaks were detected at *T* < 9 K (Figure 7). The enhancement of the χ_Μ_″ signal with frequency (Figure 7, right) is indicative of partially suppressed QTM in the ground exchange multiplet of the complex, while its rise with lowering temperature testifies to a small energy barrier for the magnetization reversal.

Hence, we tentatively deduced the relaxation parameters, *U*_eff_ and τ_0_, by applying the combined Kramers–Kronig equations (eq 3),^44^ where ω is the angular frequency, τ_0_ is the pre-exponential factor, *U*_eff_ is the effective energy barrier for the magnetization reversal, and *k*_B_ is the Boltzmann’s constant
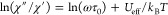
3

The best-fit parameters obtained for complex **1** (Figure S14) were: *U*_eff_ = 6.2(1) cm^–1^ [8.9(1) K] and τ_0_ = 7.5(2) × 10^–6^ s, consistent with the expected τ_0_ values for a fast-relaxing SMM. The experimental curves in Figure S14 deviate appreciably by the linear dependence of ln(χ″/χ′) vs 1/*T*, as indicated by eq 3, suggesting that a simple, single-barrier activation mechanism of relaxation is not realized in **1**. This is also confirmed by ab initio calculations (vide infra) which do not predict excited exchange states with energies close to the extracted value of *U*_eff_.

### 3.4 Single-Crystal Magnetic Hysteresis Studies

**Figure 8:**
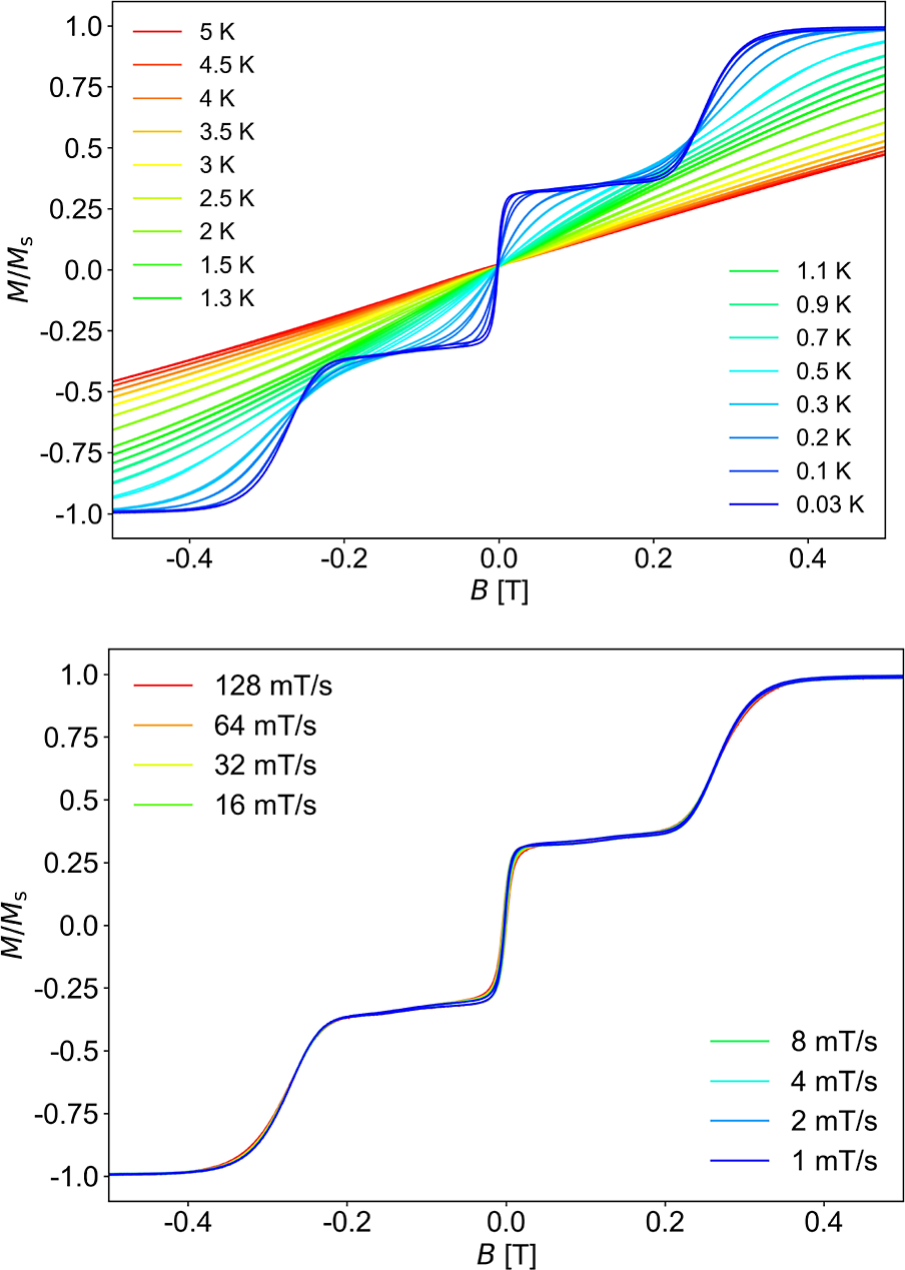
Magnetization (*M*) vs dc field hysteresis loops for a single crystal of **1** at the indicated temperatures and a fixed field sweep rate of 8 mT/s (top) and at the indicated field sweep rates and a fixed temperature of 0.03 K (bottom). The magnetization is normalized to its saturation value, *M*_S_.

**Figure 9:**
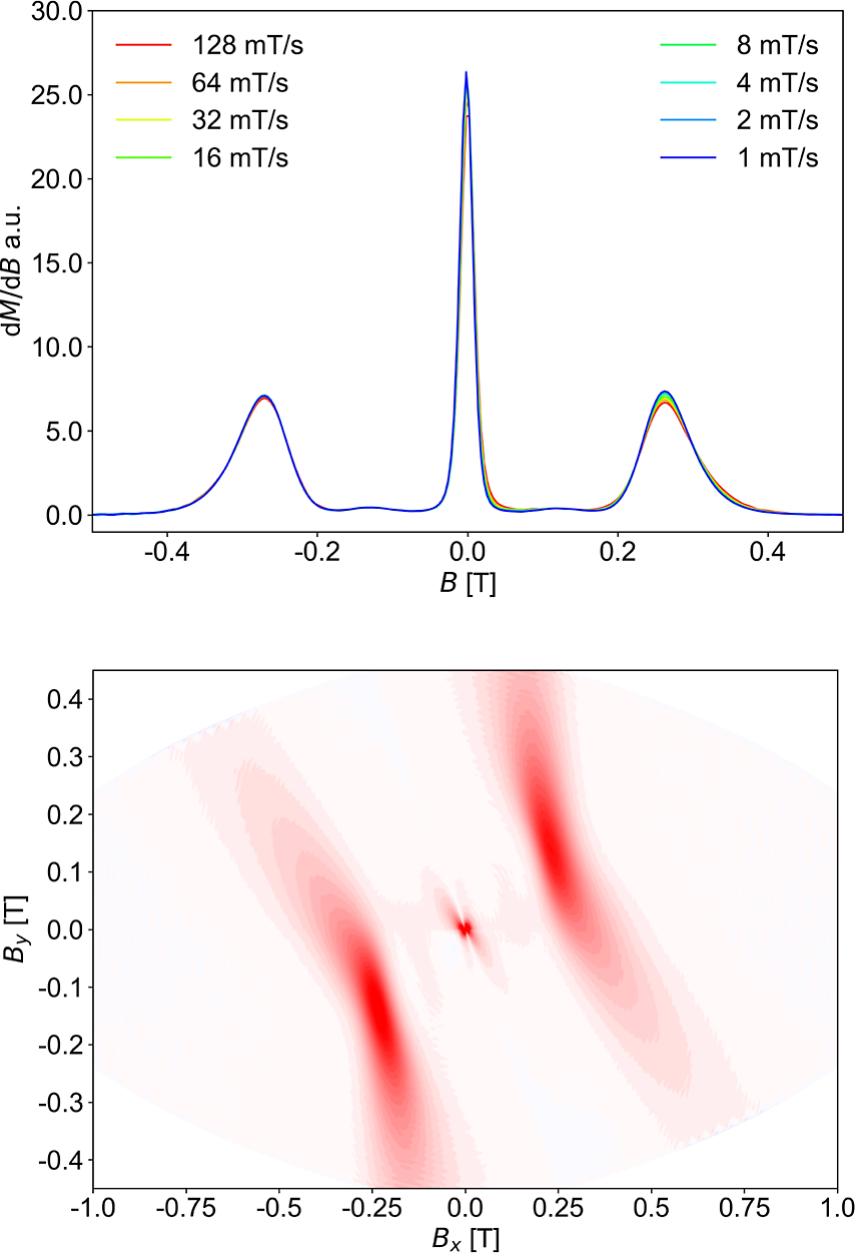
(Top) First-field derivative for a field sweep from −1.4 T to +1.4 T of the single-crystal magnetic data in Figure 8 for **1**. (Bottom) Field-angle-dependent derivative (d*M*/d*H*) map obtained from *M*(*H*) measured at different directions of field with respect to the crystal of **1**.

To assess the magnetization dynamics and better understand the mechanism of magnetization relaxation in **1**, magnetization versus applied dc field (*M*(*H*)) studies were performed on a single crystal of the {Dy_3_Bi_8_} cluster at temperatures down to 0.03 K and different field-sweep rates using a μ-SQUID apparatus. The obtained *M*(*H*) curves are shown in Figure 8, with the field applied along the easy axis of the crystal.^45^ At 30 mK temperature, the *M*(*H*) loops exhibit an *S*-shape with a rare two-step profile hysteresis cycle manifested at zero and ±0.26 T fields, respectively, as confirmed by the angular dependence of d*M*/d*H*(*H*) (Figures 9 and S15). This behavior is expected for an antiferromagnetically coupled system of three Dy^III^ atoms.^46^ In particular, the first step at the zero field, in which the magnetization rises sharply, is due to the insufficient axiality of the ground exchange doublet, while the second, less sharp step, whose profile is not influenced by the field sweep rate (Figure 8) is due to several Zeeman level crossings, as established by ab initio analysis. The temperature-dependent curves (Figure 8, top) show that these sharp features, bearing the signature of the crossings in the Zeeman diagram, disappear above a 300 mK temperature. On the other hand, the 30 mK measurements (Figure 8, bottom) clearly show that the steps are nearly sweep rate independent. The latter indicates large tunnel gaps allowing fast QTM and/or small energy separation between the consecutive states in the ground multiplet allowing for an efficient temperature-dependent relaxation pathway.

Additionally, these sharp steps in the μ-SQUID measurements have allowed us to evaluate the average Ising-type interaction (*J*_Ising_) between the Dy^III^ centers, as revealed by the inflection points between ±0.26 T.^47^ The value of the magnetic field at the crossing at ±0.26 T can be used for the calculation of *J*_Ising_ between Dy···Dy pairs in the {Dy_3_} triangle of **1**, according to the general Ising Hamiltonian: *Ĥ*_ex_ = −*J*_Ising_*s̃*_1*z*_*s̃*_2*z*_, where *s̃*_1*z*_ = *s̃*_2*z*_ = 1/2 are the pseudospins of the ground KD on each Dy site which encompasses the three {Dy_2_} edges of the triangle.^48^ The average coupling strength, *J*_Ising_, which accounts for the three Dy···Dy interactions within the equilateral {Dy_3_} triangle of **1**, can be calculated using *J*_Ising_ = −4 μ_B_*g*_J_*m*_J_*H*_ex_, where *H*_ex_ = 0.26 T, *m*_J_ = 15/2, *g*_J_ = 4/3, and μ_B_ is the Bohr magneton, giving *J*_Ising_ = −4.86 cm^–1^.^49^ Furthermore, Paul, Ungur, and Tang have recently shown that *J*_Ising_ is related to the *J*_Lines_ through the equation: *J*_Lines_ = *J*_Ising_/25, for *m*_J1_ = *m*_J2_ = 15/2.^49^ This gives an average *J*_Lines_ of −0.19 cm^–1^ for the Dy···Dy pairs in the {Dy_3_} triangle of **1**, in excellent agreement with the average value of the exchange coupling constants obtained by the ab initio calculations (vide infra).

### 3.5 Ab Initio Studies

To gain insight into the magnetic anisotropy and further understand the relaxation mechanism in **1**, ab initio calculations have been performed on the entire {Dy_3_Bi_8_} cluster based on the X-ray-determined structure (Bi^III^ ions are diamagnetic). Mononuclear Dy^III^ fragments have been included in the calculation of the CASSCF/RASSI/SINGLE_ANISO type with the OpenMOLCAS program package, in which the other two Dy^III^ ions were computationally substituted by diamagnetic Lu^III^ ions while keeping the ligand frame intact. The DZP basis set was employed on each Dy^III^ ion. As shown in Table 2, the lowest spin–orbit states (*M*_J_ = ± 15/2) of each Dy^III^ ion are well separated from the excited states. The ground doublet of each Dy^III^ ion features axial anisotropy with a *g*_*z*_ ≫ *g*_*x*,*y*_, albeit the transverse component is already relatively large for the Dy3 center preventing it alone to be an SMM. The first (and following) excited-state doublets for all three Dy^III^ ions exhibit significant contributions from the transverse components (Table 2). The energy gap between the ground doublet and the first excited state is more than 90.2 cm^–1^ for each Dy^III^ ion (Figure S16) meaning that these states do not contribute to magnetic relaxation at a very low temperature at which magnetization measurements (Figure 8) have been performed.

**Table 2:** Ab Initio Energies (cm^–1^) and *g* Factors of the Lowest Kramers Doublets (KDs) for Each Dy^III^ Ion in **1**

KD		Dy1		Dy2		Dy3	
		*E*	*g*	*E*	*g*	*E*	*g*
**1**	*g*_*x*_	0.0	0.01	0.0	0.02	0.0	0.06
	*g*_*y*_		0.02		0.03		0.13
	*g*_*z*_		19.79		19.57		19.26
**2**	*g*_*x*_	178.2	1.22	90.2	0.14	129.9	0.64
	*g*_*y*_		3.81		0.17		0.96
	*g*_*z*_		14.88		16.91		15.78
**3**	*g*_*x*_	222.3	0.05	211.4	1.72	240.1	3.43
	*g*_*y*_		3.39		2.15		4.38
	*g*_*z*_		10.82		13.16		11.56
**4**	*g*_*x*_	301.0	9.09	286.2	4.44	310.5	8.33
	*g*_*y*_		6.35		5.91		5.86
	*g*_*z*_		3.21		7.66		1.62
**5**	*g*_*x*_	372.7	1.91	332.0	1.93	382.2	0.77
	*g*_*y*_		2.45		4.27		3.78
	*g*_*z*_		11.70		12.47		11.34
**6**	*g*_*x*_	467.5	0.09	359.5	0.13	443.0	1.60
	*g*_*y*_		0.25		1.57		1.88
	*g*_*z*_		14.80		13.24		14.42
**7**	*g*_*x*_	551.7	0.10	410.7	0.17	529.7	0.30
	*g*_*y*_		0.11		0.47		0.48
	*g*_*z*_		17.66		17.80		16.91
**8**	*g*_*x*_	693.5	0.00	454.5	0.08	730.6	0.01
	*g*_*y*_		0.01		0.76		0.02
	*g*_*z*_		19.55		18.53		19.76

**Figure 10:**
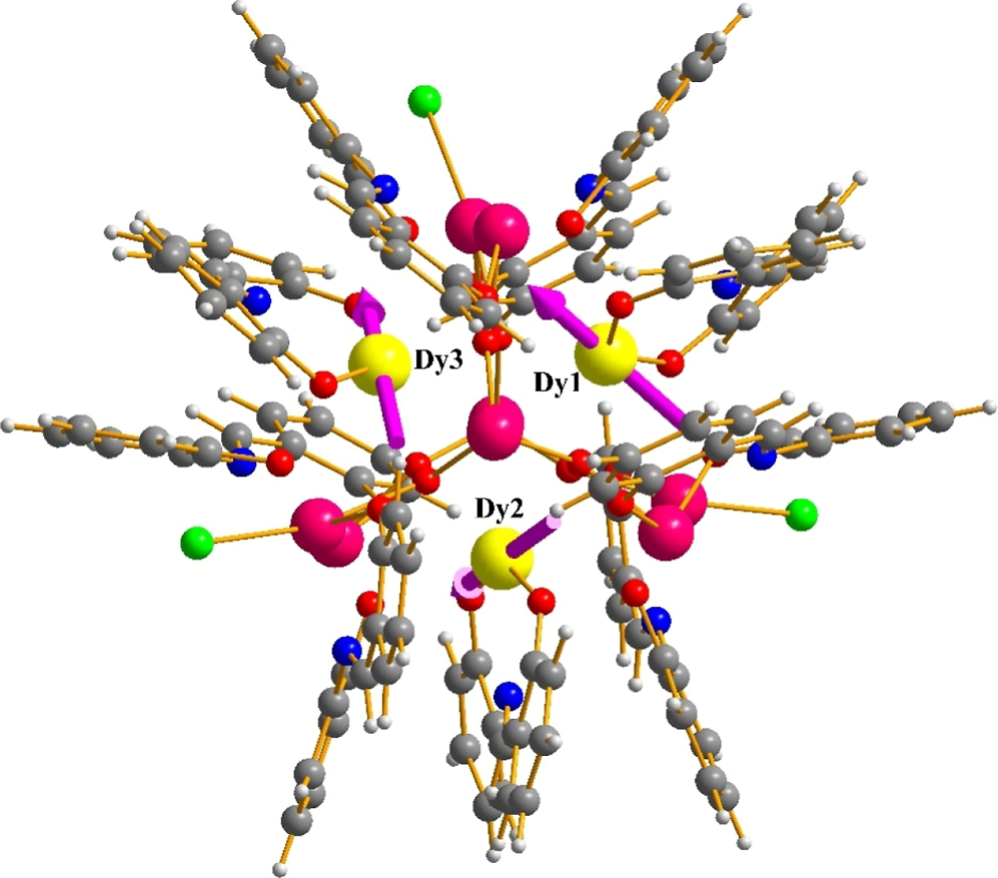
Orientations of the main magnetic axes (pink arrows) in the ground state of the {Dy_3_} unit within **1**. Only a selection of coordinated ligands is shown to better highlight the anisotropy axes. Color scheme: Dy^III^, yellow; Bi^III^, magenta; Cl, green; O, red; N, blue; C, dark gray; and H, gray.

The calculated main magnetic axes of the Dy^III^ ions are shown in Figure 10. One can see that the anisotropy axes are not arranged toroidally, deviating significantly from the plane of the {Dy_3_} triangle,^50^ thus rendering the {Dy_3_} triangle of **1** a rare example of a {Dy_3_} triangle that does not exhibit toroidicity.^51^ This can be rationalized in terms of lack of central μ_3_-bridging X-groups (X = O^2–^/OH^–^/RO^–^); in the majority of previous examples of {Dy_3_(μ_3_-Χ)} triangles, the main magnetic axes of the Dy^III^ ions are basically directed by the central bridging group in a symmetric, in-plane fashion, thus allowing for their toroidal arrangement.^52^ In the {Dy_3_} triangle of **1**, the anisotropic axes are randomly distributed and mostly pointed toward the phenolate O atoms of the coordinated saph^2–^ ligands, which are the shortest and strongest Dy–O bonds within **1**.

**Figure 11:**
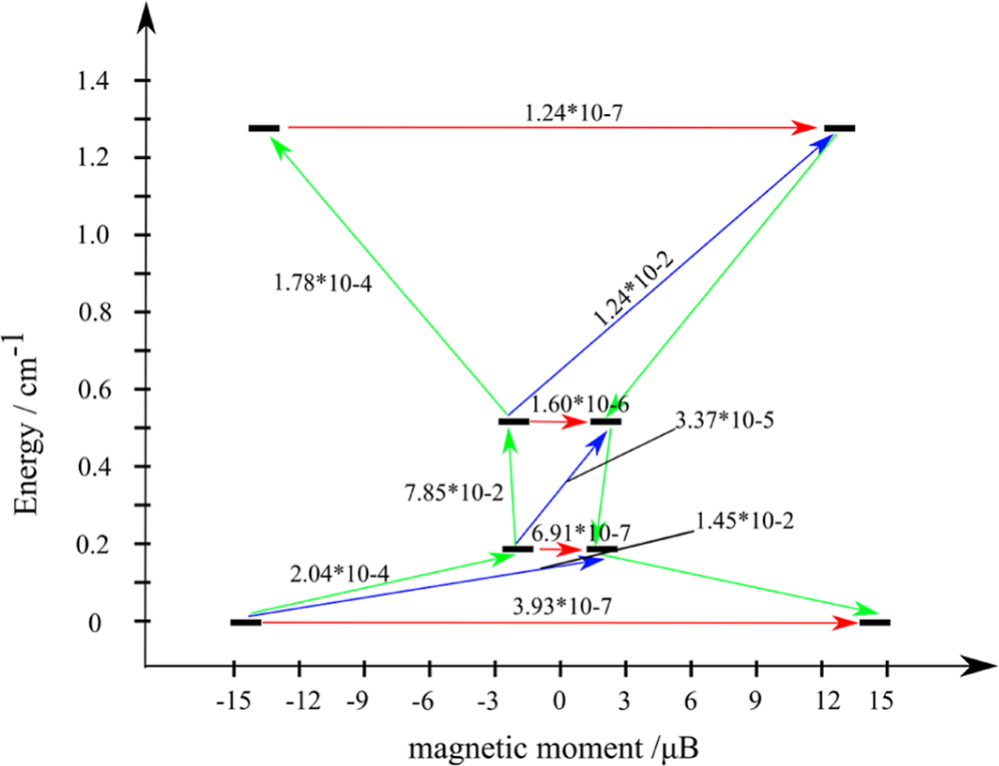
Magnetic relaxation between the exchange states in complex **1**. The red arrows with the corresponding values show the tunneling gap between the exchange states, while green and blue arrows (and values) indicate the Orbach and Orbach/Raman relaxation processes, respectively.

The magnetic interactions between Dy^III^ ions include contributions from the magnetic dipole–dipole and exchange interactions. The exchange coupling was simulated within the Lines model as described elsewhere.^29^ Projected on the ground Kramers doublets on the dysprosium sites, the magnetic interaction of the three interacting Dy^III^ ions can be described by a noncollinear Ising Hamiltonian with three individual constants (*J*_ij_), according to eq 4
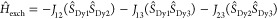
4where *s*_*i*_ is projection operators of the effective spin of the Dy^III^ ion on the corresponding anisotropy axes. The *J*_*ij*_ values are the only fitting parameters of the employed model. The intersite magnetic dipole–dipole interactions were computed using eq 5
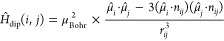
5where μ_*i*_ and μ_*j*_ are the vectors of the magnetic moments on the *i* and *j* sites, respectively, as obtained from the SINGLE_ANISO single-site calculations, *n*_*ij*_ is the normalized vector connecting sites *i* and *j* (of length = 1), *r*_*ij*_ is the distance between sites *i* and *j*, while μ_Bohr_ is the Bohr magneton. The total Hamiltonian of the magnetic interaction is given by *Ĥ*_total_ = *Ĥ*_exch_ + *Ĥ*_dip_, and the best-fit Lines parameters for the exchange interactions, recalculated for the effective spin 1/2 on the Dy sites, are listed in Table 3. The exchange interactions between the three Dy^III^ ions turned to be weak, thus inducing a very weak splitting of the resulting exchange states (Figure 11), which rationalizes the fast relaxation processes and the absence of well-resolved out-of-phase signals.^53^ At the same time, the transition magnetic moment of the exchange coupled ground state was computed to be rather weak (∼10^–7^, red arrows in Figure 11), the same for the other three low-lying exchange doublets, thus ruling out the QTM as a basic mechanism of magnetic relaxation. However, the transition magnetic moment is relatively large across different energy states (10^–2^ between the ground and the first excited doublets) paving the way for efficient one-phonon (Orbach) and possible two-phonon (Raman) relaxation pathways (green and blue arrows in Figure 11).^54^ At temperatures of the order or exceeding the energy of the first excited KD of complex **1** (0.2 cm^–1^), the temperature dependence of the one-phonon relaxation rate will not be of the exponential type, which is confirmed by the nonlinear Debye plots shown in Figure S14. On the other hand, the existence of nonvanishing out-of-phase signals in the ac susceptibility is proof of partially suppressed QTM in the ground exchange doublet. Both of these facts are confirmed by the ab initio calculations.

**Table 3:** Exchange Coupling and Dipolar Interactions (in cm^–1^) Calculated for **1**^a^^,^^b^

interaction	Dy1–Dy2	Dy1–Dy3	Dy2–Dy3
exchange coupling	–0.39	–0.21	0.02
dipolar interactions	0.91	1.14	–1.62
total magnetic interactions	0.52	0.93	–1.60

a The exchange coupling parameters were obtained within the Lines model and then written in the Ising model.

b For the dipolar interactions, only the *zz* component is considered but all terms were included in the POLY_ANISO calculations.

**Figure 12:**
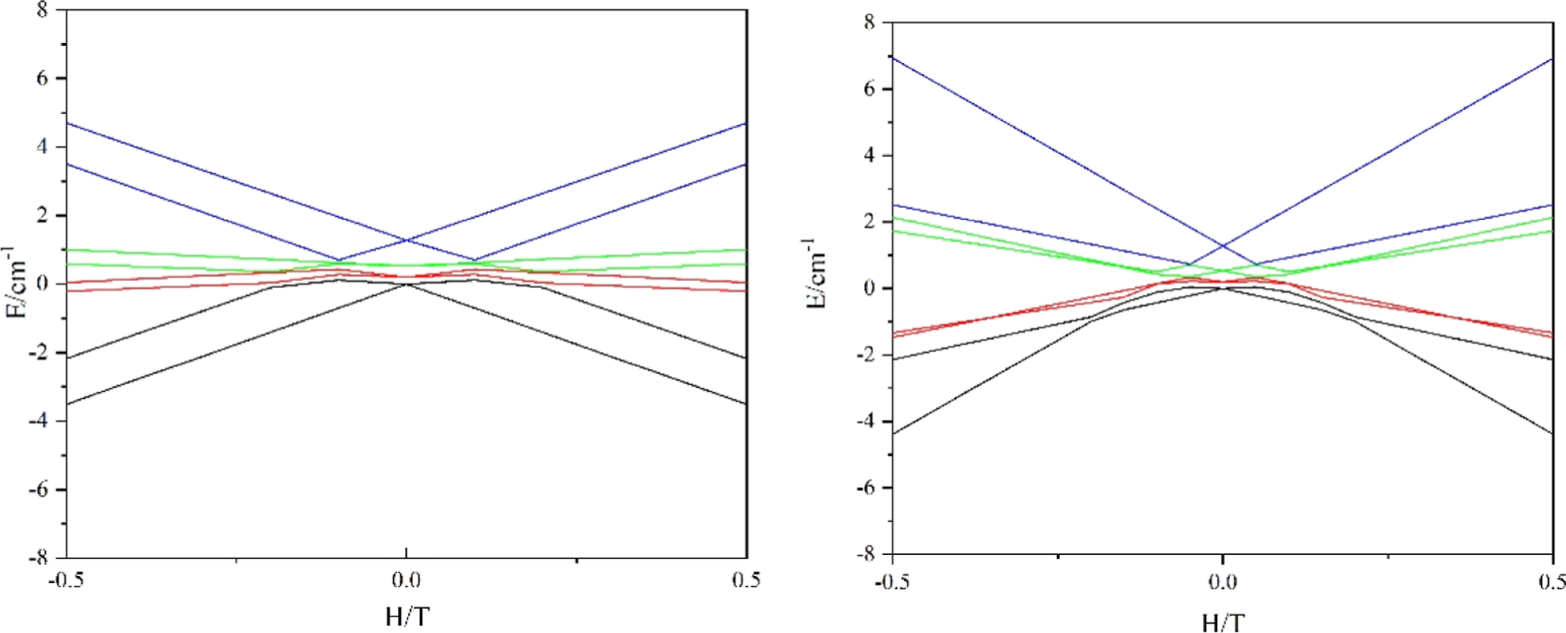
Calculated evolution of the lowest eight exchange states of **1** with the applied field. The field is applied along the main anisotropy axis of the first (right) and fourth (left) exchange doublets in complex **1**.

**Figure 13:**
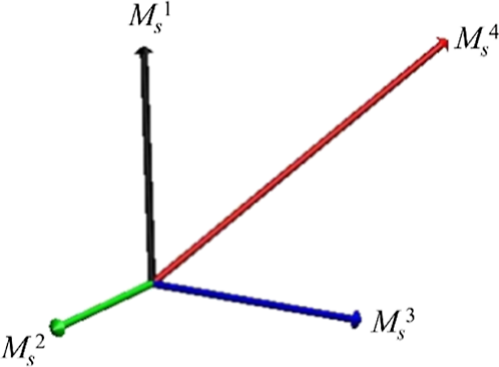
Main magnetic axes of the four low-lying exchange doublets in **1**.

The left plot in Figure 12 shows a Zeeman diagram of the lowest four exchange KDs of **1** for an external field applied along the main magnetic axis of the ground KD. Given its relatively large *g*-factor (Table 4) and the fact that the main magnetic axes of the other three exchange KDs make an appreciable angle with the former (Figure 13), the ground doublet is never intersected by the Zeeman components of the excited doublet for such a direction of the applied field. This is, however, not what is observed in experiment suggesting crossing of Zeeman components at 0.26 T. The explanation of this puzzle comes from a significantly larger *g*-factor (and saturation moment) in the highest exchange doublet (Table 4). The right plot in Figure 12 shows that when the field is applied along the main magnetic axis of the fourth exchange KD, a level crossing occurs at ∼0.2 T. The discrepancy with the experimental value can arise from underestimated excitation energy of the fourth exchange doublet (1.3 cm^–1^) and/or from not exact alignment of the applied field to the direction of its main magnetic axis.

**Table 4:** Ab Initio Energies (cm^–1^) and *g* Tensors of the Lowest Four Exchange Doublets in **1**

exchange doublets		*E*	*g*
**1**	*g*_*x*_	0.0	0.00
	*g*_*y*_		0.00
	*g*_*z*_		30.09
**2**	*g*_*x*_	0.2	0.00
	*g*_*y*_		0.00
	*g*_*z*_		21.25
**3**	*g*_*x*_	0.5	0.00
	*g*_*y*_		0.00
	*g*_*z*_		29.54
**4**	*g*_*x*_	1.3	0.00
	*g*_*y*_		0.00
	*g*_*z*_		48.50

## 4 Conclusions

In conclusion, we have reported the first heterometallic Bi^III^/Dy^III^ complexes, consisting of the molecular cluster **1** and its 1-D analogue **2**, through the targeted replacement of terminal Cl^–^ ions by end-to-end bridging N_3_^–^ groups. Both compounds are supported by bridging O^2–^ ions and the RO^–^ arms of the Schiff base saph^2–^, forming an overall unique metallic topology, which comprises a {Bi_8_} unit with an elongated trigonal bipyramidal topology surrounding a {Dy_3_} equilateral triangle. Interestingly, the coordination geometries of the Bi^III^ centers revealed some vacant coordination sites, indicative of the arrangement of the lone pair of bismuth 6s electrons. In addition to the interesting structural features, complex **1** exhibits relaxation of magnetization, albeit with a small energy barrier due to the onset of fast quantum tunneling. Detailed magnetic hysteresis studies at 30 mK on a single crystal of **1** revealed an *S*-shape hysteresis loop with a rare two-step profile at zero and ±0.26 T fields, providing a measure of intermolecular Dy···Dy interactions. Finally, ab initio calculations were in support of the experimental magnetic studies, shading light into the anisotropy of the individual Dy^III^ ions, the exchange interactions, and the mechanisms of magnetization relaxation, which render the {Dy_3_} unit of **1** as a rare triangle with a nontoroidal magnetic state. The absence of SMM behavior in this compound turns out to be not the lack of appreciable axial magnetic anisotropy on the three Dy sites and in the low-lying exchange doublets but rather the small energy separation between the latter and the efficient one-phonon relaxation pathway between the ground and the first excited exchange doublet.

We are currently aiming to expand the heterometallic Ln/post-transition metal chemistry (i.e., Dy/Bi, Dy/Sn, Dy/Ga, and Dy/In) as a means of obtaining molecular magnetic compounds (with or without metal–metal bonds) with enhanced magnetization dynamics induced by the contribution of the heavy diamagnetic metal to the total SOC (heavy ion effect) and subsequently to the overall magnetic anisotropy.

## Supporting Information Available

Bond distances and angles, IR and powder XRD plots, structural and supramolecular figures, magnetism plots, and some details for the theoretical calculations performed on complexes **1** and **2** (PDF)
