# Supplementary material for: Unveiling the Werner-Type Cluster Chemistry of Heterometallic 4f/Post-Transition Metals: A {Dy3Bi8} Complex Exhibiting Quantum Tunneling Steps in the Hysteresis Loops and its 1-D Congener
Source: Inorg Chem. 2025 Jan 17;64(4):1962–74. doi: 10.1021/acs.inorgchem.4c04721 (PMC11795526; doi:10.1021/acs.inorgchem.4c04721)
Supplement: Supplementary file 1 — ic4c04721_si_001.pdf [file ic4c04721_si_001.pdf]

# ELECTRONIC SUPPORTING INFORMATION

## Unveiling the Werner-type cluster chemistry of heterometallic 4f/post-transition metals: A {$Dy_3Bi_8$} complex exhibiting quantum tunneling steps in the hysteresis loops, and its 1-D congener

**Konstantina H. Baka,[a] Dan Liu,[b] Sagar Paul,[c] Wolfgang Wernsdorfer,[c,d] Jinkui Tang,[e] Liviu F. Chibotaru,*[,f] Theocharis C. Stamatatos*[,a,g]**

[a] Department of Chemistry, University of Patras, 265 04 Patras, Greece
[b] School of Science, Changchun Institute of Technology, Changchun 130012, P. R. China
[c] Physikalisches Institut, Karlsruhe Institute of Technology (KIT), Kaiserstraße 12, Karlsruhe, D-76131, Germany
[d] Institute for Quantum Materials and Technology (IQMT), Karlsruhe Institute of Technology (KIT), Hermann-von-Helmholtz-Platz 1, Eggenstein-Leopoldshafen, D-76344, Germany
[e] State Key Laboratory of Rare Earth Resource Utilization, Changchun Institute of Applied Chemistry, Chinese Academy of Sciences, Changchun 130022, P. R. China
[f] Theory of Nanomaterials Group, Katholieke Universiteit Leuven, Celestijnenlaan 200F, Leuven, B-3001, Belgium
[g] Institute of Chemical Engineering Sciences, Foundation for Research and Technology – Hellas (FORTH/ICE – HT), Platani, P.O. Box 1414, 26504 Patras, Greece

Corresponding authors: Prof. Theocharis C. Stamatatos – Prof. Liviu F. Chibotaru
E-mails: thstama@upatras.gr & liviu.chibotaru@gmail.com

**Table S1.** Contractions of the employed basis set in computational approximations for complex **1**.

| Basis set |
|---|
| Dy. ANO-RCC-VDZP |
| Lu. ANO-RCC-VDZ |
| N. ANO-RCC-VDZ |
| O. ANO-RCC-VDZ |

**Table S2.** Selected bond distances (Å) and angles (°) for complex **1**.

| **Bond distances (Å)** | | | |
|---|---|---|---|
| Dy1-O1 | 2.410(7) | Bi3-Cl2 | 2.918(5) |
| Dy1-O6 | 2.400(6) | Bi3-O8 | 2.170(6) |
| Dy1-O8 | 2.575(7) | Bi3-O11 | 2.525(8) |
| Dy1-O11 | 2.376(7) | Bi3-O15 | 2.193(8) |
| Dy1-O12 | 2.586(6) | Bi3-O16 | 2.430(7) |
| Dy1-O17 | 2.325(8) | Bi3-O20 | 2.81(1) |
| Dy1-O18 | 2.693(8) | Bi3-N6 | 2.394(9) |
| Dy1-O20 | 2.463(7) | Bi4-O3 | 2.632(8) |
| Dy1-N7 | 2.512(13) | Bi4-O4 | 2.108(6) |
| Dy2-O1 | 2.652(6) | Bi4-O5 | 2.645(8) |
| Dy2-O3 | 2.529(8) | Bi4-O10 | 2.317(7) |
| Dy2-O4 | 2.629(6) | Bi4-O24 | 2.231(8) |
| Dy2-O5 | 2.273(7) | Bi4-N1 | 2.418(9) |
| Dy2-O7 | 2.511(7) | Bi5-Cl4 | 2.778(4) |
| Dy2-O12 | 2.410(7) | Bi5-O7 | 2.479(9) |
| Dy2-O14 | 2.405(7) | Bi5-O12 | 2.194(6) |
| Dy2-O21 | 2.308(8) | Bi5-O17 | 2.503(9) |
| Dy2-N9 | 2.496(13) | Bi5-O18 | 2.780(9) |
| Dy3-O4 | 2.430(6) | Bi5-O23 | 2.200(9) |
| Dy3-O6 | 2.603(7) | Bi5-N5 | 2.398(10) |

| | | | |
|---|---|---|---|
| Dy3-O8 | 2.402(6) | Bi6-O1 | 2.180(7) |
| Dy3-O9 | 2.333(8) | Bi6-O8 | 2.131(7) |
| Dy3-O10 | 2.592(7) | Bi6-O13 | 2.858(8) |
| Dy3-O14 | 2.584(7) | Bi6-O14 | 2.112(6) |
| Dy3-O16 | 2.505(7) | Bi6-O15 | 2.713(8) |
| Dy3-O22 | 2.335(8) | Bi6-O19 | 2.694(9) |
| Dy3-N2 | 2.512(12) | Bi7-Cl3 | 2.892(5) |
| Bi1-O2 | 2.719(9) | Bi7-O2 | 2.190(7) |
| Bi1-O4 | 2.151(7) | Bi7-O6 | 2.164(6) |
| Bi1-O6 | 2.141(6) | Bi7-O16 | 2.822(9) |
| Bi1-O12 | 2.134(7) | Bi7-O20 | 2.457(7) |
| Bi1-O23 | 2.631(9) | Bi7-O22 | 2.537(8) |
| Bi1-O24 | 2.797(8) | Bi7-N4 | 2.368(10) |
| Bi2-Cl1 | 2.795(4) | Bi8-O1 | 2.088(7) |
| Bi2-O3 | 2.526(7) | Bi8-O7 | 2.737(8) |
| Bi2-O9 | 2.556(8) | Bi8-O13 | 2.226(8) |
| Bi2-O10 | 2.896(7) | Bi8-O18 | 2.300(7) |
| Bi2-O14 | 2.233(7) | Bi8-O21 | 2.565(8) |
| Bi2-O19 | 2.174(7) | Bi8-N10 | 2.421(8) |
| Bi2-N8 | 2.394(11) | | |
| **Bond angles (°)** | | | |
| Dy1-O1-Dy2 | 109.6(2) | Dy1-O8-Dy3 | 110.2(2) |
| Dy1-O12-Dy2 | 111.9(3) | Dy2-O4-Dy3 | 109.8(2) |
| Dy1-O6-Dy3 | 109.3(2) | Dy2-O14-Dy3 | 112.1(3) |

**Table S3.** Selected bond distances (Å) and angles (°) for complex **2**.

| **Bond distances (Å)** | | | |
|---|---|---|---|
| Dy1-O1 | 2.525(7) | Bi3-O18 | 2.80(1) |

| | |
|---|---|
| Dy1-O8 | 2.550(7) |
| Dy1-O11 | 2.297(11) |
| Dy1-O13 | 2.346(7) |
| Dy1-O14 | 2.320(11) |
| Dy1-O19 | 2.355(7) |
| Dy1-O20 | 2.544(8) |
| Dy1-O21 | 2.556(8) |
| Dy1-N3 | 2.49(3) |
| Dy2-O1 | 2.343(7) |
| Dy2-O7 | 2.554(9) |
| Dy2-O12 | 2.398(9) |
| Dy2-O15 | 2.316(10) |
| Dy2-O16 | 2.577(8) |
| Dy2-O18 | 2.530(8) |
| Dy2-O19 | 2.560(8) |
| Dy2-O22 | 2.319(10) |
| Dy2-N2 | 2.54(3) |
| Dy3-O3 | 2.288(9) |
| Dy3-O4 | 2.590(8) |
| Dy3-O6 | 2.320(11) |
| Dy3-O12 | 2.613(8) |
| Dy3-O13 | 2.544(7) |
| Dy3-O16 | 2.327(8) |
| Dy3-O20 | 2.389(8) |
| Dy3-O24 | 2.457(8) |
| Dy3-N1 | 2.50(3) |
| Bi1-O1 | 2.152(7) |
| Bi1-O2 | 2.688(9) |
| Bi1-O9 | 2.737(9) |
| Bi3-O19 | 2.185(7) |
| Bi3-O21 | 2.376(9) |
| Bi3-O22 | 2.553(9) |
| Bi3-N7 | 2.786(9) |
| Bi3-N11 | 2.355(11) |
| Bi4-O1 | 2.188(7) |
| Bi4-O10 | 2.193(10) |
| Bi4-O14 | 2.474(9) |
| Bi4-O18 | 2.436(8) |
| Bi4-O21 | 2.771(9) |
| Bi4-N10 | 2.418(12) |
| Bi4-N15 | 2.661(13) |
| Bi5-O4 | 2.745(9) |
| Bi5-O6 | 2.515(10) |
| Bi5-O7 | 2.389(12) |
| Bi5-O12 | 2.116(7) |
| Bi5-O17 | 2.200(13) |
| Bi5-N17 | 2.377(12) |
| Bi6-O3 | 2.655(9) |
| Bi6-O8 | 2.357(8) |
| Bi6-O9 | 2.174(9) |
| Bi6-O13 | 2.155(7) |
| Bi6-O24 | 2.747(8) |
| Bi6-N16 | 2.388(10) |
| Bi7-O8 | 2.738(8) |
| Bi7-O11 | 2.570(11) |
| Bi7-O20 | 2.170(8) |
| Bi7-O23 | 2.168(12) |
| Bi7-O24 | 2.444(9) |

| | | | |
|---|---|---|---|
| Bi1-O10 | 2.699(9) | Bi7-N4A | 2.90(4) |
| Bi1-O13 | 2.152(8) | Bi7-N8 | 2.370(15) |
| Bi1-O16 | 2.168(8) | Bi8-O2 | 2.185(11) |
| Bi2-O5 | 2.728(10) | Bi8-O4 | 2.388(9) |
| Bi2-O12 | 2.152(8) | Bi8-O7 | 2.774(9) |
| Bi2-O17 | 2.77(1) | Bi8-O15 | 2.559(9) |
| Bi2-O19 | 2.121(9) | Bi8-O16 | 2.161(7) |
| Bi2-O20 | 2.130(7) | Bi8-N12 | 2.392(10) |
| Bi2-O23 | 2.674(14) | Bi8-N14 | 2.735(14) |
| Bi3-O5 | 2.193(12) | | |
| **Bond angles (°)** | | | |
| Dy1-O1-Dy2 | 111.0(3) | Dy1-O20-Dy3 | 109.7(3) |
| Dy1-O19-Dy2 | 109.4(3) | Dy2-O12-Dy3 | 108.4(3) |
| Dy1-O13-Dy3 | 111.1(3) | Dy2-O16-Dy3 | 111.9(3) |

**Table S4.** Continuous Shape Measures (CShM) values for the potential coordination polyhedra of the 9-coordinate $Dy^{III}$ centers in the structure of complex **1**.

| **Polyhedron*a,b*** | **Dy1** | **Dy2** | **Dy3** |
|---|---|---|---|
| EP-9 | 34.74 | 33.70 | 35.01 |
| OPY-9 | 22.36 | 21.43 | 22.44 |
| HBPY-9 | 18.45 | 18.40 | 18.09 |
| JTC-9 | 16.69 | 16.08 | 16.74 |
| JCCU-9 | 7.33 | 7.67 | 6.81 |
| CCU-9 | 5.74 | 6.36 | 5.34 |
| JCSAPR-9 | 2.65 | 2.11 | 2.40 |
| **CSAPR-9** | **1.51** | **1.15** | **1.36** |
| JTCTPR-9 | 4.23 | 3.30 | 3.99 |
| TCTPR-9 | 1.53 | 1.39 | 1.62 |
| JTDIC-9 | 10.30 | 10.97 | 10.81 |

| | | | |
|---|---|---|---|
| HH-9 | 10.56 | 11.17 | 10.71 |
| MFF-9 | 2.25 | 1.80 | 2.12 |

[a] Abbreviations: EP-9, Enneagon; OPY-9, Octagonal pyramid; HBPY-9, Heptagonal bipyramid; JTC-9, Johnson triangular cupola; JCCU-9, Capped cube; CCU-9, Spherical-relaxed capped cube; JCSAPR-9, Capped square antiprism, CSAPR-9, Spherical capped square antiprism; JTCTPR-9, Tricapped trigonal prism; TCTPR-9, Spherical tricapped trigonal prism; JTDIC-9, Tridiminished icosahedron, HH-9, Hula-hoop; MFF-9, Muffin. [b] The values in boldface indicate the closest polyhedron according to the Continuous Shape Measures.

**Table S5.** Continuous Shape Measures (CShM) values for the potential coordination polyhedra of the 6- and 7-coordinate $Bi^{III}$ centers in the structure of complex **1**.

| **6-coordinate $Bi^{III}$ centers** | | | | |
|---|---|---|---|---|
| **Polyhedron[a,b]** | **Bi1** | **Bi4** | **Bi6** | **Bi8** |
| HP-6 | 40.42 | 24.89 | 40.28 | 25.09 |
| PPY-6 | 18.07 | 4.55 | 18.45 | 4.52 |
| OC-6 | 17.94 | 23.42 | 15.47 | 24.57 |
| **TPR-6** | **5.67** | 13.34 | **5.56** | 13.55 |
| **JPPY-6** | 22.65 | **3.92** | 23.32 | **3.84** |
| **7-coordinate $Bi^{III}$ centers** | | | | |
| **Polyhedron[a,b]** | **Bi2** | **Bi3** | **Bi5** | **Bi7** |
| HP-7 | 29.19 | 29.77 | 29.36 | 28.61 |
| HPY-7 | 22.62 | 22.57 | 22.05 | 22.95 |
| PBPY-7 | 6.50 | 6.42 | 7.60 | 8.10 |
| COC-7 | 6.02 | 7.00 | 6.15 | 6.83 |
| **CTPR-7** | **4.11** | **4.96** | **4.26** | **4.53** |
| JPBPY-7 | 10.09 | 10.27 | 11.05 | 11.94 |
| JETPY-7 | 18.07 | 18.97 | 17.46 | 17.51 |

[a] Abbreviations: HP-6, Hexagon; PPY-6, Pentagonal pyramid; OC-6, Octahedron; TPR-6, Trigonal prism; JPPY-6, Johnson pentagonal pyramid J2; HP-7, Heptagon; HPY-7, Hexagonal pyramid; PBPY-7, Pentagonal bipyramid; COC-7, Capped octahedron, CTPR-7, Capped trigonal prism; JPBPY-7, Johnson pentagonal bipyramid; JETPY-7, Johnson

elongated triangular pyramid. $^{b}$ The values in boldface indicate the closest polyhedron according to the Continuous Shape Measures.

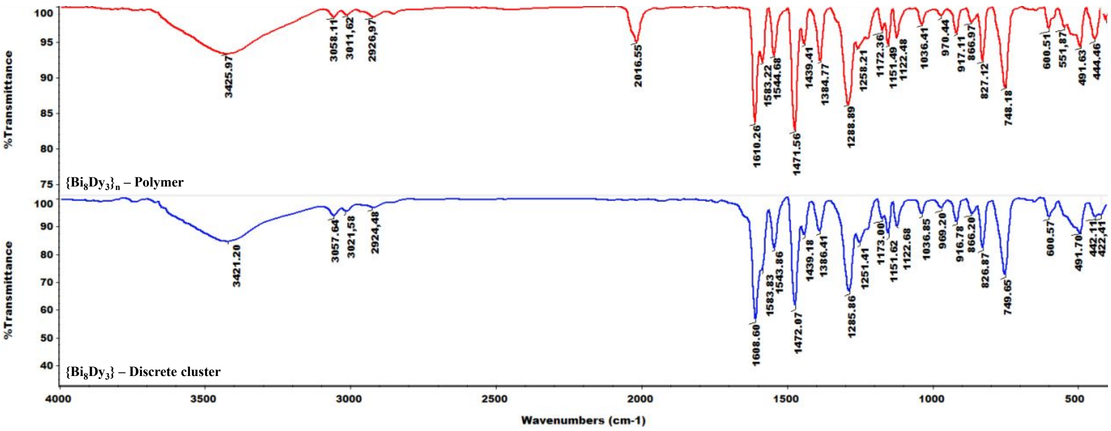


**Figure S1.** FT-IR spectra of complexes **1** (bottom) and **2** (top).

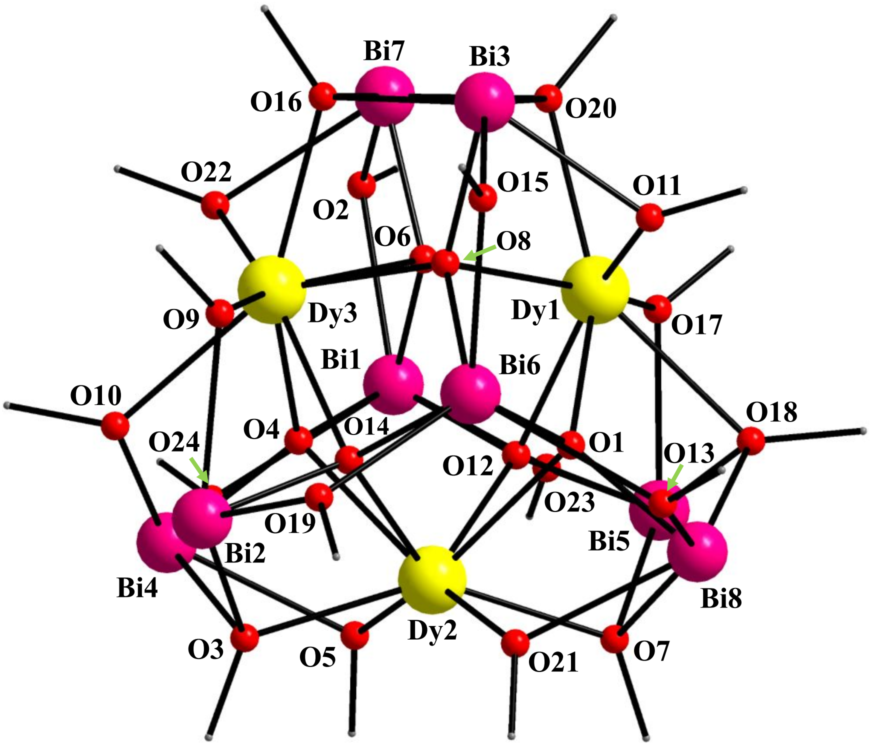


**Figure S2.** Representation of the complete $\{Dy_3Bi_8(\mu_4\text{-}O)_6(\mu_3\text{-}OR)_5(\mu\text{-}OR)_{13}\}^{3+}$ core of **1**; the $RO^-$ groups belong to the bridging phenoxido arms of $saph^{2-}$. Color scheme: $Dy^{III}$, yellow; $Bi^{III}$, magenta; Cl, green; O, red; N, blue; C, gray. H atoms are omitted for clarity.

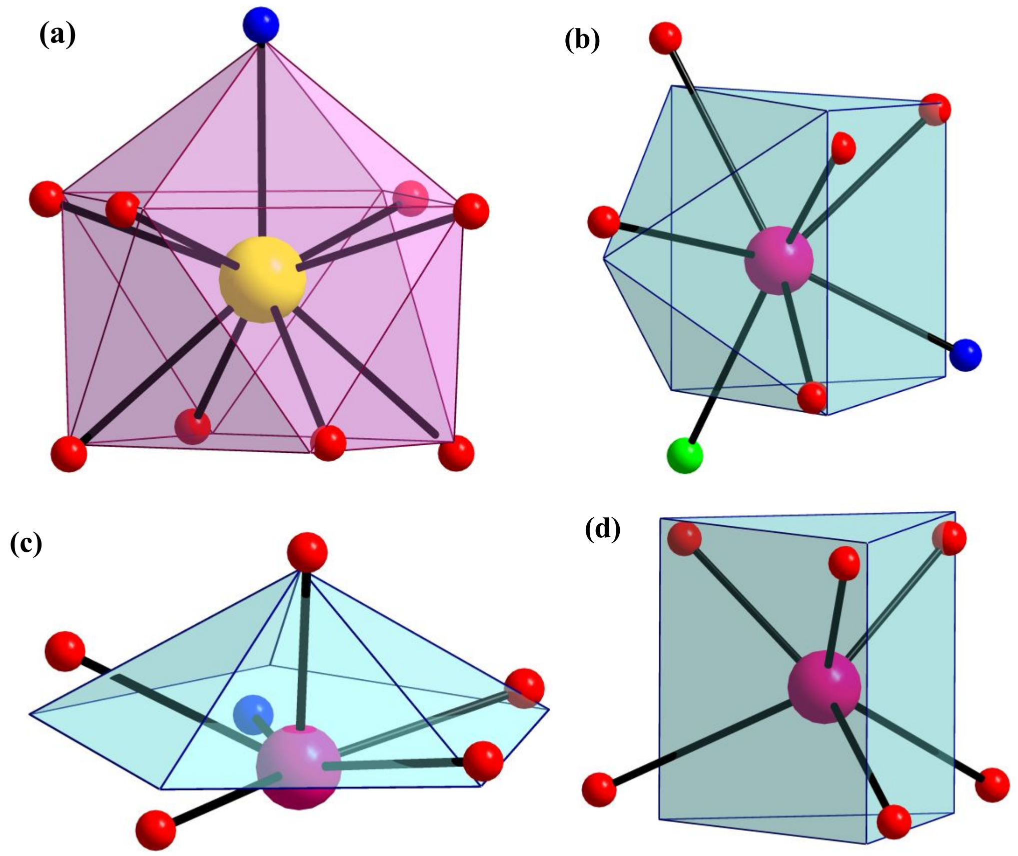


**Figure S3.** (a) Spherical capped square antiprismatic coordination geometry of the $Dy^{III}$ atoms in **1**, and (b) capped trigonal prismatic, (c) Johnson pentagonal pyramidal, and (d) trigonal prismatic coordination geometries of the $Bi^{III}$ atoms in **1**. Points connected by the gray/blue thin lines define the vertices of the ideal polyhedra. Color scheme as in Figure S2. The six-coordinate Bi(4,8) adopt a distorted Johnson pentagonal pyramidal geometry [CShM = 3.92 (Bi4) and 3.84 (Bi8)], whereas Bi(1,6) are arranged into a heavily distorted trigonal prismatic environment [CShM = 5.67 (Bi1) and 5.56 (Bi6)]; both geometries have vacant coordination sites for the $Bi^{III}$ atoms, which is a first indication for the arrangement of the lone pair of 6*s* electrons. The seven-coordinate Bi(2,3,5,7) adopt a capped trigonal prismatic geometry [CShM = 4.11 (Bi2), 4.96 (Bi3), 4.26 (Bi5) and 4.53 (Bi7)] with the capping atom being a phenoxido O-atom from a $saph^{2-}$ chelate.

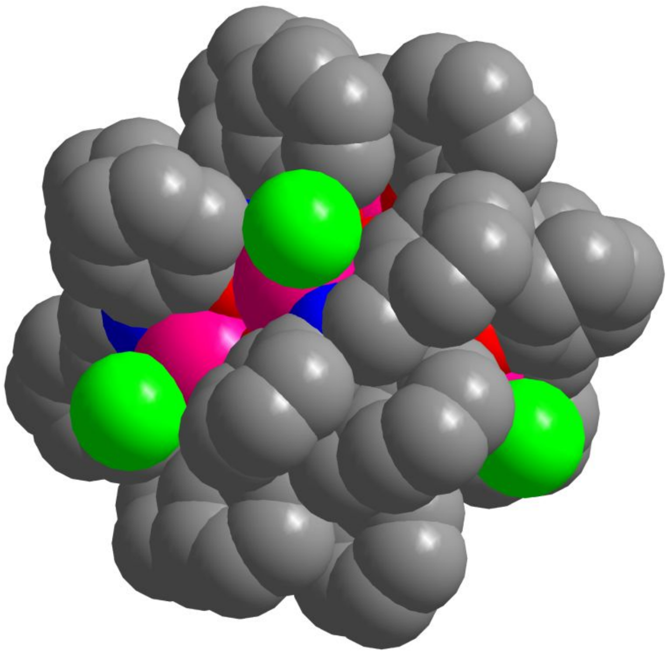

**Figure S4.** Space-filling representation of **1** viewed along the *b*-axis. Color scheme: $Dy^{III}$, yellow; $Bi^{III}$, magenta; Cl, green; O, red; N, blue; C, gray.

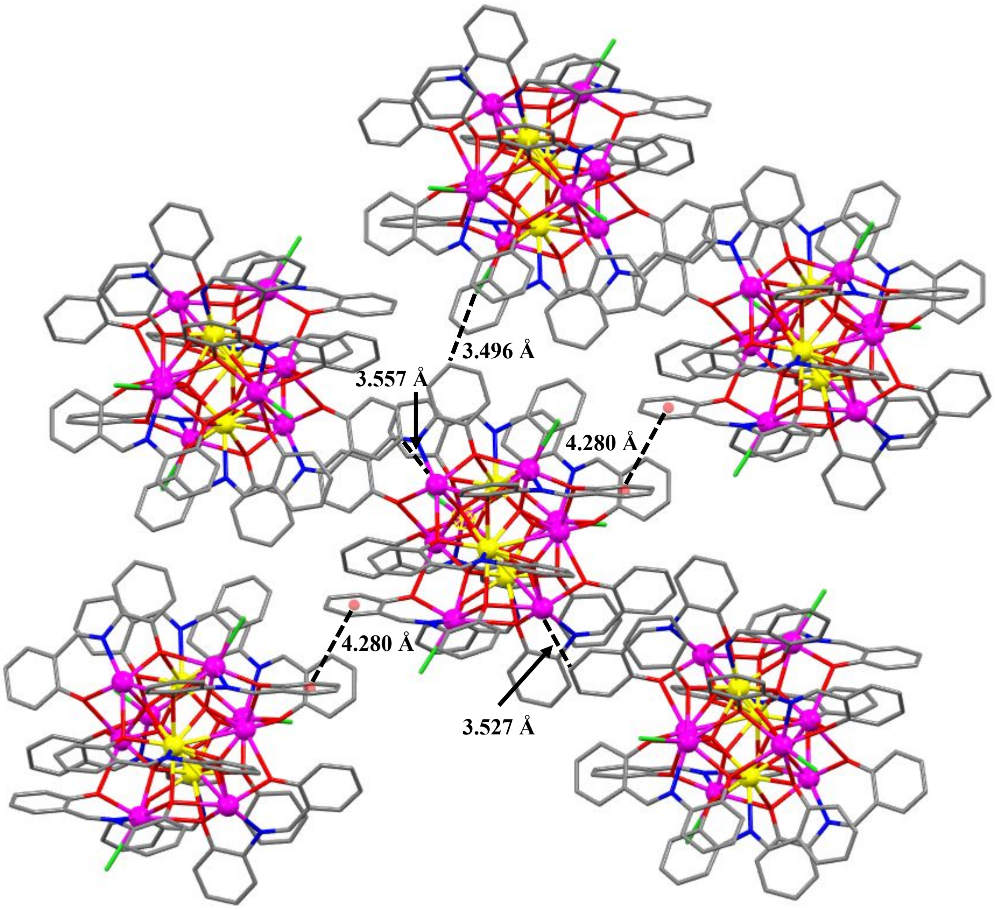


**Figure S5.** Portion of the $\{Dy_3Bi_8\}$ clusters in the crystal of **1**, highlighting their interactions (with dashed lines) with neighboring units through CH···π stacking

interactions between the phenyl substituents of the $saph^{2-}$ ligands and the aromatic rings of $saph^{2-}$ and the lone pair of electrons of $Bi^{III}$ atoms. All H atoms are omitted for clarity. Color scheme: $Dy^{III}$, yellow; $Bi^{III}$, magenta; Cl, green; O, red; N, blue; C, gray.

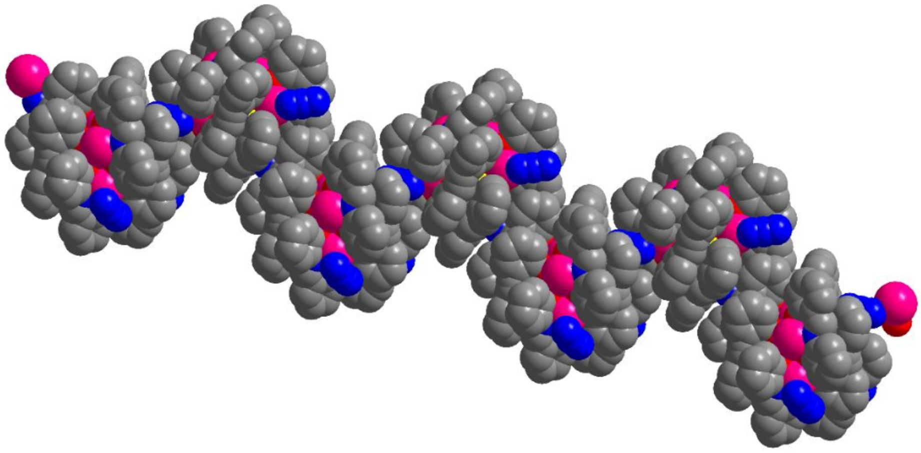

**Figure S6.** Space-filling representation of the 1-D polymer-of-clusters **2** viewed along the *ab*-plane. Color scheme: $Dy^{III}$, yellow; $Bi^{III}$, magenta; O, red; N, blue; C, gray.

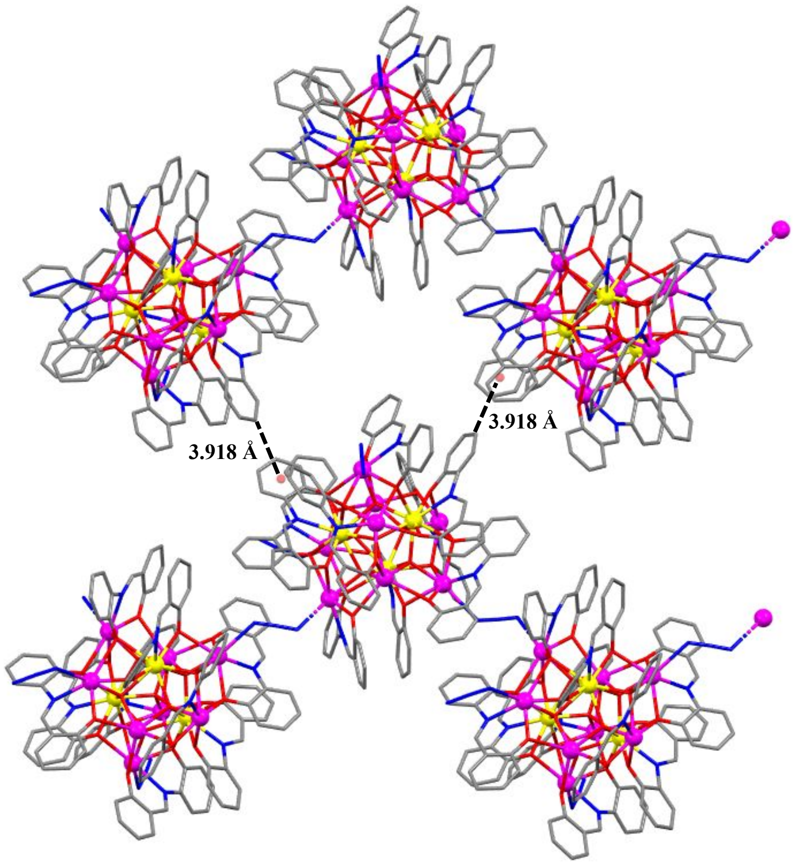


**Figure S7.** Portion of the 1-D chain of $\{Dy_3Bi_8\}$ clusters in the crystal of **2**, highlighting the interactions (with dashed lines) with adjacent chains through CH···π stacking

interactions between the phenyl substituents of the saph$^{2-}$ ligands. All H atoms are omitted for clarity. Color scheme: Dy$^{III}$, yellow; Bi$^{III}$, magenta; O, red; N, blue; C, gray.

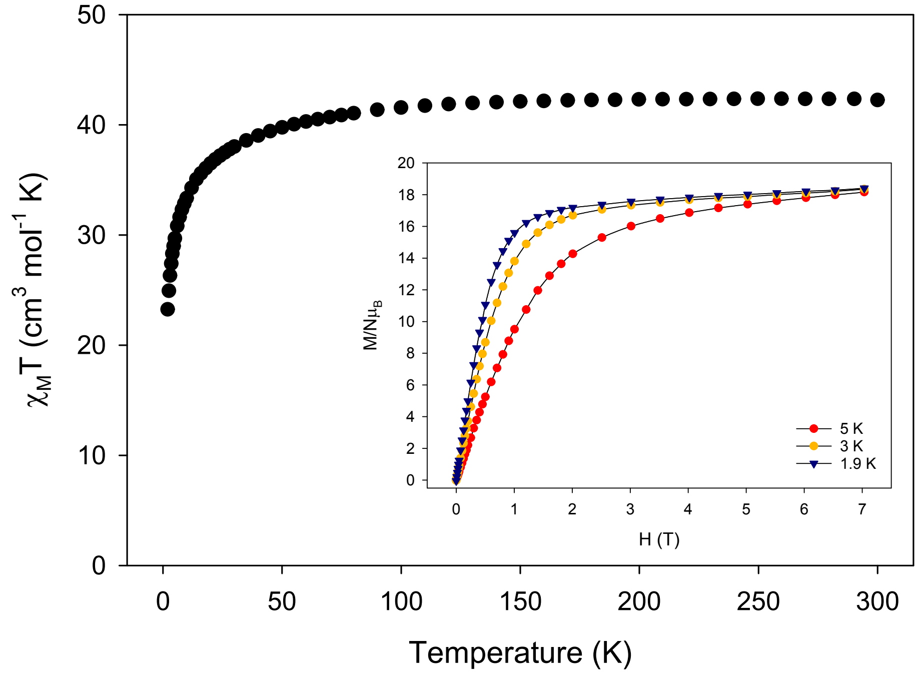


**Figure S8.** Temperature dependence of the $\chi_M T$ product for the 1-D polymer **2** at 0.1 T. (inset) Plots of magnetization (*M*) *vs.* field (*H*) for **2** at three different low temperatures. The solid lines are guides only.

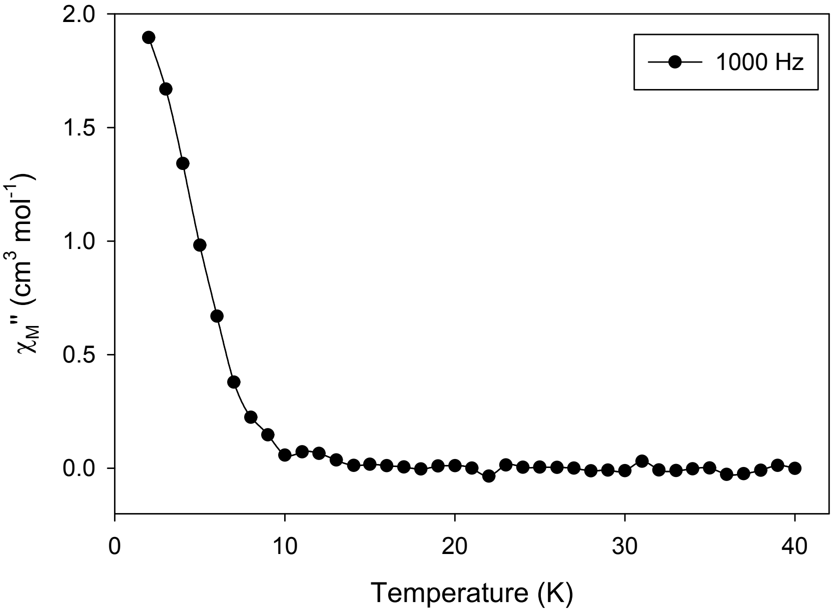


**Figure S9.** Temperature dependence of the out-of-phase ($\chi_M$'') ac magnetic susceptibility in zero dc field for the 1-D polymer **2**, measured in a 3.0 G ac field oscillating at the frequency of 1000 Hz. The solid line is guide only.

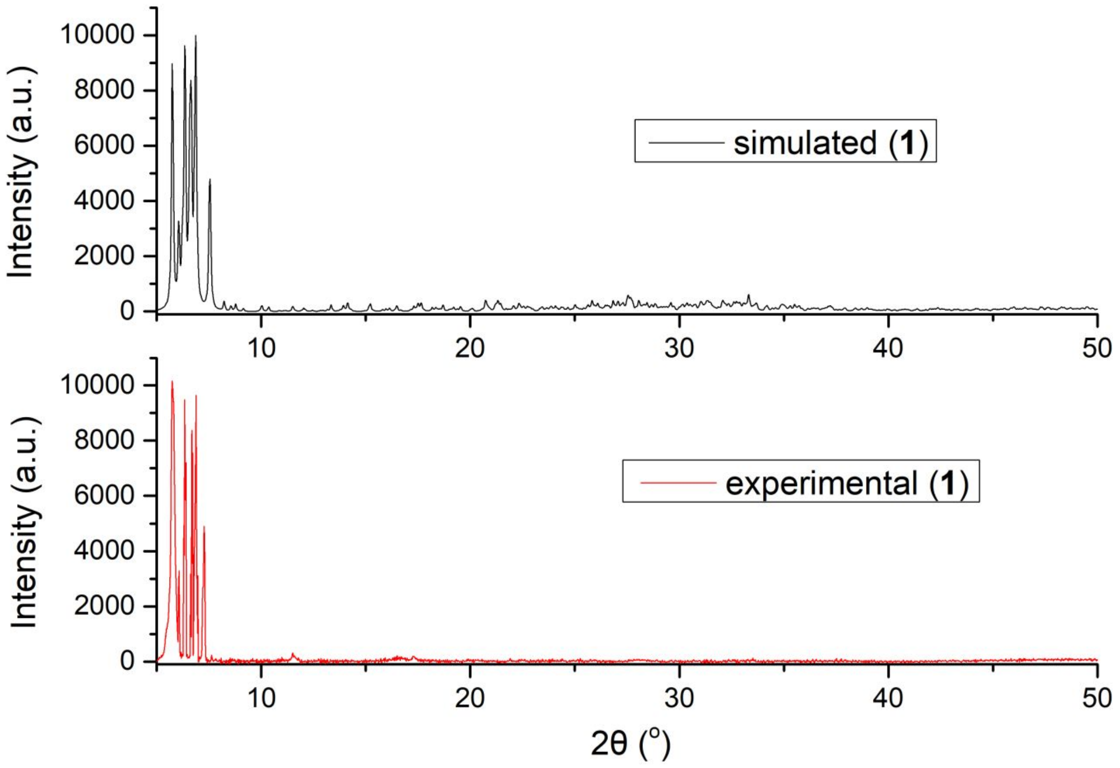


**Figure S10.** Experimental and simulated powder X-ray diffraction (p-XRD) patterns of **1**. The simulated pattern is calculated based on the structural model from the single-crystal X-ray diffraction data.

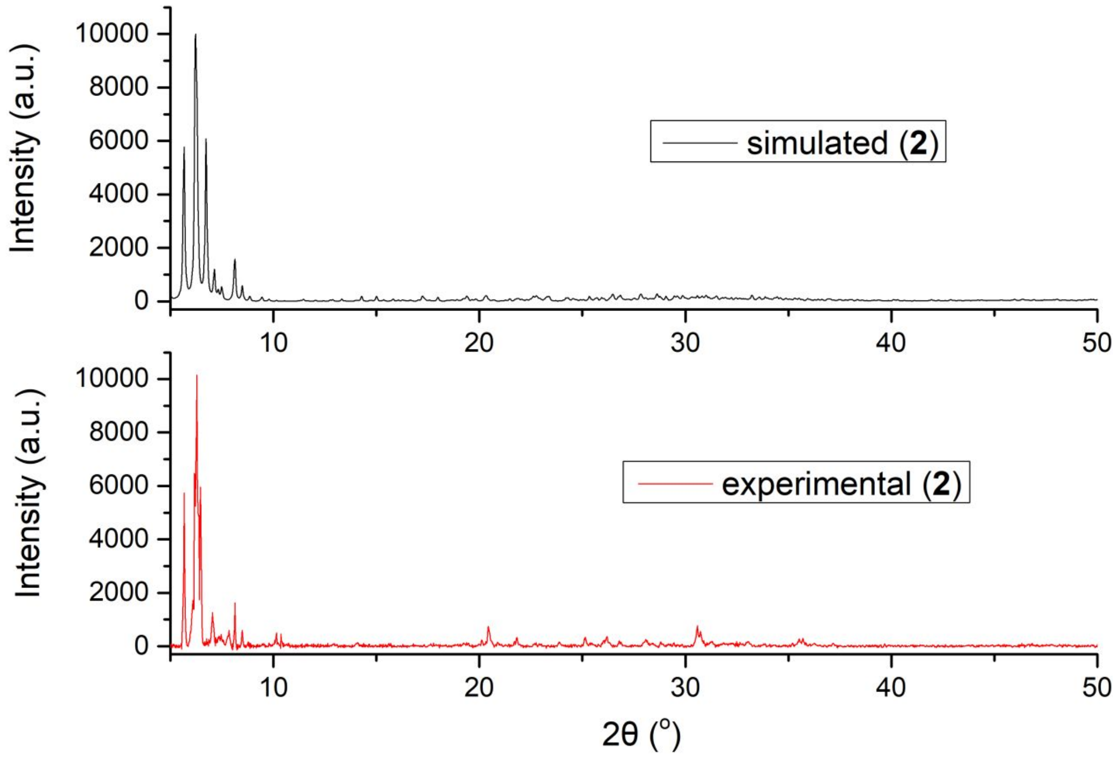

**Figure S11.** Experimental and simulated powder X-ray diffraction (p-XRD) patterns of **2**. The simulated pattern is calculated based on the structural model from the single-crystal X-ray diffraction data.

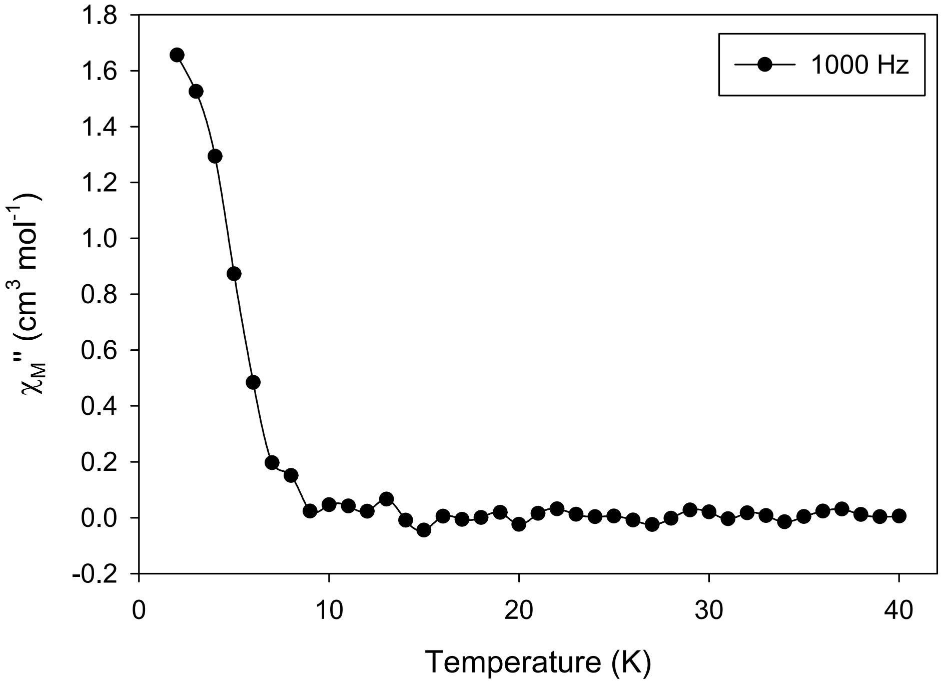


**Figure S12.** Temperature dependence of the out-of-phase ($\chi_M''$) ac magnetic susceptibility in zero dc field for **1**, measured in a 3.0 G ac field oscillating at the frequency of 1000 Hz. The solid line is guide only.

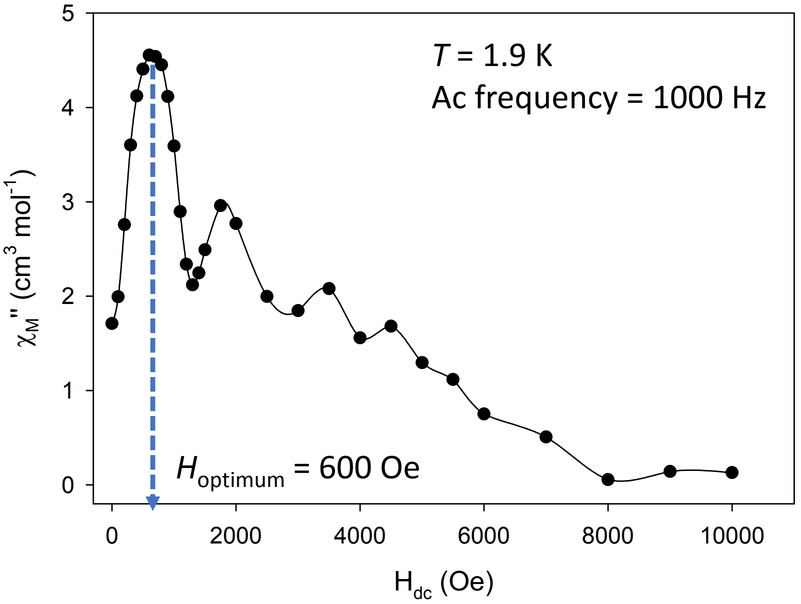

**Figure S13.** Field ($H$) dependence of the out-of-phase ($\chi_M''$) ac magnetic susceptibility of **1** measured at 1.9 K. The strongest peak maximum corresponds to the optimum dc field. The solid line is guide only.

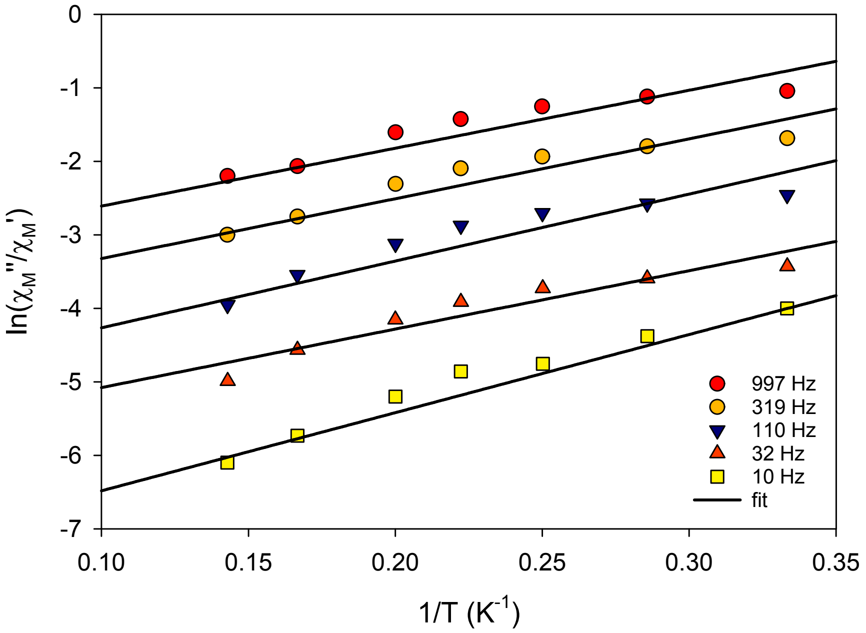


**Figure S14.** Debye plots of complex **1** for the indicated ac frequencies. The solid lines correspond to the fit of the data by applying equation (3); see the text for the fit parameters.

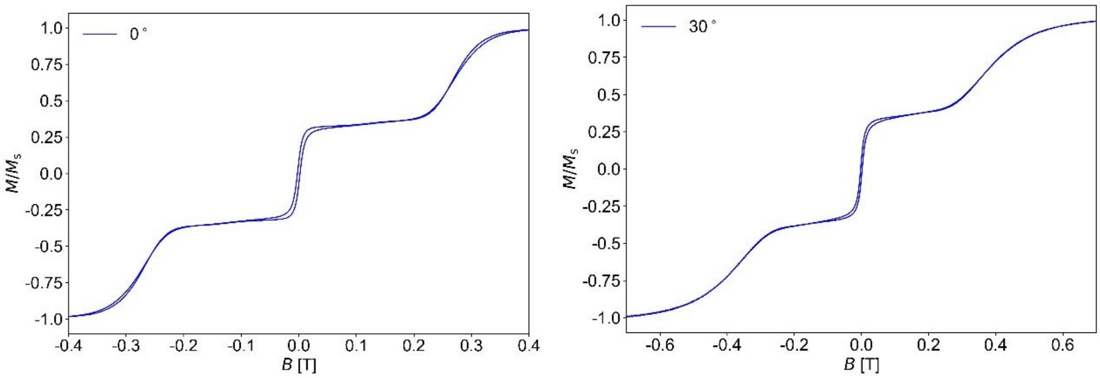

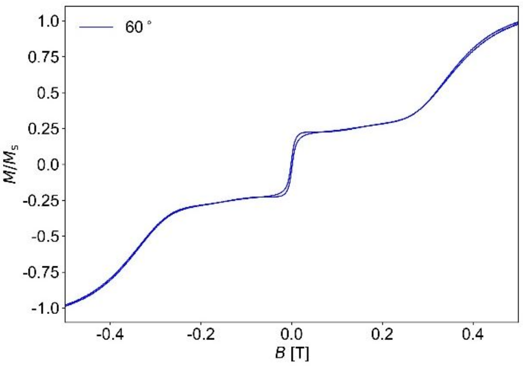


**Figure S15.** Angular dependence of the magnetic hysteresis loops of **1** at $T$ = 0.03 K. The magnetization is normalized to its saturation value, $M_S$.

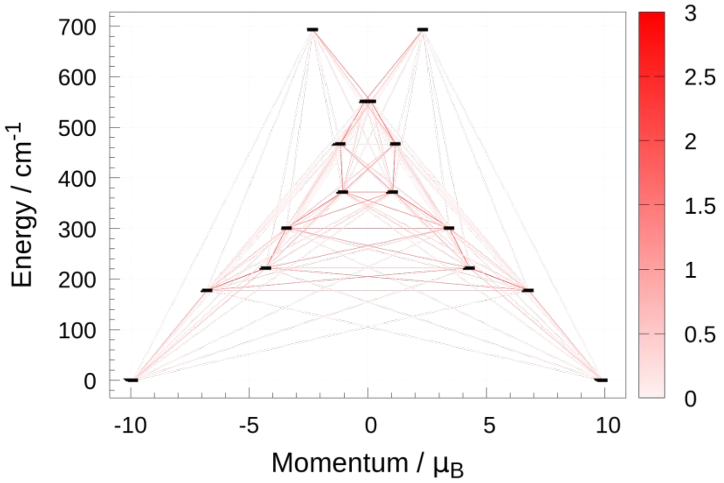


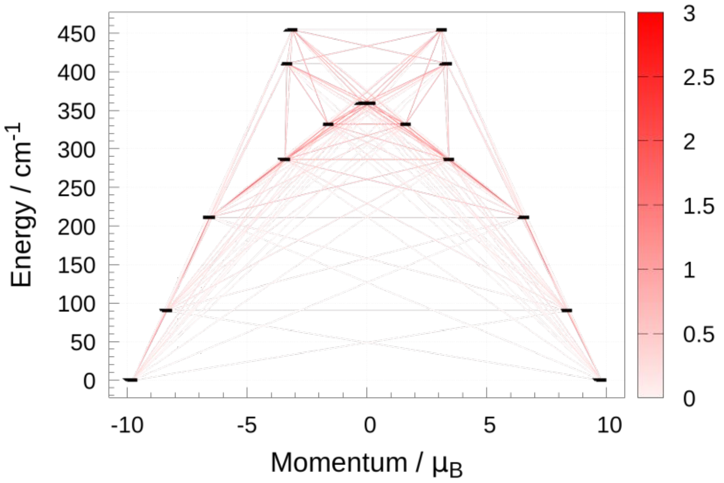

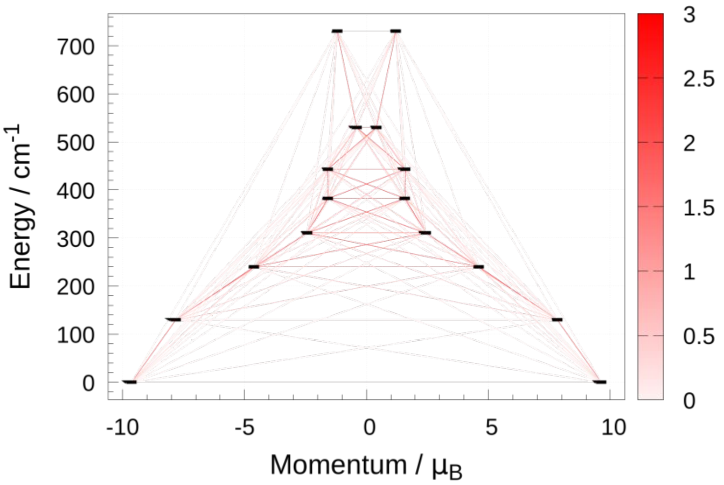


**Figure S16.** Energy (in cm$^{-1}$) *vs.* momentum for the ground *J* = 15/2 of Dy1 (top), Dy2 (middle) and Dy3 (bottom) sites. The states are placed according to the value of their magnetic moment (black thick lines). The horizontal red lines show the tunneling transitions within each doublet state, while the non-horizontal ones show the spin-phonon transition pathways. The intensity of the red lines indicates the amplitude of the average transition magnetic dipole moment in $\mu_B$ between the connected states (see the legend in the right-hand side), the square of which roughly scales with the rate of spin-phonon transition between them.
